# D3Impute: Dropout-aware discrimination, distribution-aware modeling, and density-guide imputation for scRNA-seq data

**DOI:** 10.1371/journal.pcbi.1013744

**Published:** 2025-12-01

**Authors:** Siyi Huang, Linfeng Jiang, Ming Yi, Yuan Zhu

**Affiliations:** 1 School of Mathematics and Physics, China University of Geosciences, Wuhan, Hubei, China; 2 School of Automation, China University of Geosciences, Wuhan, Hubei, China; Georgia Institute of Technology College of Computing, UNITED STATES OF AMERICA

## Abstract

Single-cell RNA sequencing (scRNA-seq) has revolutionized the study of cellular heterogeneity. A major challenge, however, lies in the prevalence of non-biological zeros—false measurements caused by technical limitations that mask a cell’s true transcriptome. This fundamental issue of distinguishing these artifacts from true biological zeros, where a gene is genuinely absent, remains a key hurdle for computational methods, as misclassification can distort biological signals during data recovery. To overcome this, we introduce D3Impute, a discriminative imputation framework built on three key innovations: (1) a distribution-aware normalization step that adapts to dataset-specific characteristics while preserving meaningful biological variation; (2) a dual-network discriminator that uses bulk RNA-seq data as a biological reference to accurately identify non-biological zeros while retaining the true biological zeros; and (3) a density-guided imputation engine that recovers expression values while maintaining local cellular neighborhood structures. Through comprehensive benchmarking against 12 state-of-the-art methods across six diverse datasets, D3Impute demonstrates consistent and significant improvements in essential downstream analyses, including cell clustering, trajectory inference, and differential expression detection. Furthermore, we provide an extensive practical evaluation of D3Impute, demonstrating its robustness across varying data qualities and providing clear guidelines for optimal application. By offering a robust, biologically informed, and user-oriented solution, D3Impute not only enhances scRNA-seq data analysis but also offers a generalizable framework for handling zero-inflated data in computational biology.

## Author summary

Single-cell RNA sequencing (scRNA-seq) reveals cellular heterogeneity but is compromised by technical “dropout" events—non-biological zeros that obscure true expression patterns. To address this, we developed D3Impute, a computational framework built on three core innovations: (1) Distribution-aware modeling adapts normalization to each cell’s statistical properties, moving beyond one-size-fits-all approaches; (2) Dropout-aware discrimination integrates cell–cell networks from scRNA-seq data with gene co-expression networks from bulk RNA-seq to accurately identify non-biological zeros; (3) Density-guided imputation employs a neighborhood-preserving algorithm with dynamic weighting to recover missing values while preventing over-smoothing and retaining meaningful cellular heterogeneity. Together, these components form a principled and interpretable framework that significantly enhances the accuracy of scRNA-seq data analysis.

## Introduction

Single-cell RNA sequencing (scRNA-seq) has revolutionized genomic research by enabling high-resolution characterization of cellular gene expression profiles, reveals tissue heterogeneity, and deciphers complex biological mechanisms [1]. However, a critical analytical challenge stems from the exceptionally high prevalence of zero values (65%-90%) in scRNA-seq data matrices [2]. These observed zero values encompass biological factors-induced missing events (true zeros) and measurement-induced technical artifacts (false zeros), constituting a substantial source of noise that significantly impacts data interpretation [3]. True zeros, also referred to as biological zeros, represent the absence of a gene’s transcripts or messenger RNAs in a cell. In contrast, false zeros, termed non-biological zeros, reflect the loss of information due to the inefficiencies of the technologies employed from sample collection to sequencing [4]. Therefore, rigorously distinguishing between biological zeros and non-biological zeros, along with developing precise imputation methodologies for dropout events, represents an essential foundational step for ensuring the reliability and biological validity of subsequent analytical workflows.

The rapid progress in single-cell transcriptomics has driven significant innovation in computational methods for scRNA-seq data imputation, yielding diverse methodological approaches. In this study, we systematically organize existing techniques through a dual-aspect classification framework (summarized in Table 1) that considers both fundamental algorithmic principles and computational architectures. At the algorithmic level, we categorize them into: model-driven methods that incorporate explicit biological assumptions about gene expression distributions; data smoothing techniques that leverage local similarity patterns across cellular or gene neighborhoods; deep learning approaches utilizing neural networks to capture complex expression relationships; and hybrid frameworks that strategically combine multiple paradigms to enhance performance. Architecturally, these methods can be distinguished by their implementation strategy, falling into two categories: Direct imputation and Indirect imputation.

**Table 1:** Overview of scRNA-seq imputation methods.

Method	Category	Code Sources	Language
MAGIC (2018) [5]	H (Algorithm integration) /*N*_1_	https://github.com/KrishnaswamyLab/MAGIC	Python/R
DrImpute (2018) [6]	S (Cell)/*N*_2_	https://github.com/ikwak2/DrImpute	R
scRMD (2018) [7]	M (Low-rank)/*N*_1_	https://github.com/XiDsLab/scRMD	R
scVI (2018) [8]	H (Algorithm integration) /*N*_1_	https://github.com/YosefLab/scVI	Python
scImpute (2018) [9]	M (Gamma+Normal) /*N*_2_	https://github.com/Vivianstats/scImpute	R
netSmooth (2018) [10]	H (Data integration) /*N*_1_	https://github.com/BIMSBbioinfo/netSmooth	R
SAVER (2018) [11]	M (NB) /*N*_1_	https://github.com/mohuangx/SAVER	R
SAVER-X (2019) [12]	M (NB+) /*N*_1_	https://github.com/jingshuw/SAVERX	R
SCRABBLE (2019) [13]	H (Data integration) /*N*_1_	https://github.com/tanlabcode/SCRABBLE	R/MATLAB
DCA (2019) [14]	D (Autoencoders) /*N*_1_	https://github.com/theislab/dca	Python
bayNorm (2020) [15]	M (Bayesian) /*N*_1_	https://github.com/WT215/bayNorm	R
CMFImpute (2020) [16]	M (low-rank) /*N*_2_	https://github.com/xujunlin123/CMFImpute	Python
SIMPLEs (2020) [17]	S (Cell) /*N*_2_	https://github.com/JunLiuLab/SIMPLEs2020	R
VIPER (2020) [18]	H (Algorithm integration) /*N*_2_	https://github.com/ChenMengjie/Vpaper2018	R
G2S3 (2021) [19]	S (Gene) /Y	https://github.com/ZWang-Lab/G2S3	MATLAB/R
scGNN (2021) [20]	D (GNN+Autoencoder) /Y	https://github.com/juexinwang/scGNN	Python
SDImpute (2021) [21]	S (Cell) /*N*_2_	https://github.com/Jinsl-lab/SDImpute	R
scTSSR (2021) [22]	S (Gene and Cell)/Y	https://github.com/Zhangxf-ccnu/scTSSR	Python
ALRA (2022) [23]	M (Low-rank) /*N*_2_	https://github.com/KlugerLab/ALRA	R
GE-Impute (2022) [24]	S (Cell) /*N*_1_	https://github.com/wxbCaterpillar/GE-Impute	Python
scBERT (2022) [25]	D (Transformer) /Y	https://github.com/TencentAILabHealthcare/scBERT	Python
GraphSCI (2022) [26]	D (GNN+Autoencoder) /Y	https://github.com/biomed-AI/GraphSCI	Python
scRNMF (2023) [27]	M (Low-rank) /*N*_1_	https://github.com/QYuQing/scRNMF	R
scGGAN (2023) [28]	H (Algorithm integration) /*N*_1_	https://www.sdu-idea.cn/codes.php?name=scGGAN	Python
TsImpute (2023) [29]	H (Algorithm integration) /*N*_2_	https://github.com/ZhengWeihuaYNU/tsImpute	R
scAMF (2024) [30]	S (Manifold) /Y	https://github.com/zhigang-yao/scAMF	MATLAB
AGImpute (2024) [31]	H (Algorithm integration) /*N*_2_	https://github.com/xszhu-lab/AGImpute	Python

S: data smoothing methods, M: model-driven methods, D: deep learning methods, H: hybrid methods, Y: direct imputation, *N*_1_: explicit or implicit differentiation and imputation, *N*_2_: two-stage predict-then-impute approaches. The methods marked with horizontal lines are the 12 state-of-the-art approaches selected for benchmarking in our comparative analysis.

**(1) Model-driven methods**

Model-driven approaches operate on the fundamental assumption that scRNA-seq data matrices exhibit inherent low-rank structures. These methods employ matrix factorization techniques to reconstruct missing values, with notable implementations including scRMD (single-cell Robust Matrix Decomposition) [7], ALRA (Adaptively thresholded Low-Rank Approximation) [23], CMFImpute (Collaborative Matrix Factorization Imputation) [16], scRNMF (single-cell Robust and Non-negative Matrix Factorization) [27], and others.

Alternatively, a distinct subset of model-driven methods incorporates explicit probabilistic frameworks, modeling gene expression through specific distributions (e.g., Poisson or Negative Binomial). These methods estimate missing values by fitting model parameters to the observed data, as exemplified by SAVER/SAVER-X (Single-cell Analysis Via Expression Recovery) [11,12], bayNorm (bayesian Normalization) [15], scImpute (single-cell Imputation) [9], and others. While particularly effective at capturing global transcriptional patterns, these methods face inherent limitations when handling ultra-sparse datasets, as excessive sparsity may violate core model assumptions, ultimately compromising imputation accuracy.

**(2) Data smoothing methods**

Data smoothing approaches impute missing values by exploiting similarities between cells, genes, or their combined patterns via similarity calculation or manifold fitting. Representative methods include DrImpute (Dropout Imputation) [6], SIMPLEs (SIngle-cell RNA-seq iMPutation and celL clustErings) [17], scAMF (single-cell Analysis via Manifold Fitting) [30], GE-Impute (Graph Embedding-based Imputation) [24], G2S3 (Sparse Gene Graph of Smooth Signals) [19], scTSSR (scRNA-seq using a Two-side Sparse Self-Representation) [22], SDImpute (Single-cell RNA-seq Dropout Imputation) [21], and others proposed methods for imputing dropout events considering cell-level correlation. Data smoothing techniques for imputing single-cell RNA sequencing data offer benefits such as reducing data sparsity, improving consistency, and enhancing downstream analysis performance. However, these methods may introduce bias by replacing genuine biological zeros with non-zero values. Additionally, reliance on specific model assumptions can increase computational complexity, thereby hindering data processing efficiency.

**(3) Deep learning methods**

Deep learning has emerged as a powerful paradigm for scRNA-seq imputation, with four predominant architectures: (1) autoencoders (e.g., DCA [14]); (2) generative adversarial networks (e.g., scGGAN [28]); (3) graph neural networks (e.g., GraphSCI [26], scGNN [20]), and (4) Transformer models (e.g., scBERT [25]). Implementation requires careful consideration of three critical factors: computational resource requirements, scalability to large datasets, and preservation of biologically meaningful patterns in the imputed data.

**(4) Hybrid methods**

In addition to the methods mentioned above, several approaches integrate multiple techniques or combine multi-omics and multi-modal data to enhance imputation accuracy. For example, VIPER (Variability-preserving ImPutation for Expression Recovery) [18] combines low-rank matrix factorization with cell similarity metrics, effectively merging model-based and similarity-based approaches. scGGAN [28] integrates generative adversarial networks (GANs) with gene similarity metrics and generates dropout values to impute the raw scRNA-seq data. Similarly, MAGIC (Markov Affinity-based Graph Imputation of Cells) [5] employs diffusion processes and low-dimensional embedding techniques to exploit the manifold structure of the data for imputation. AGImpute [31] combines probabilistic dropout modeling with an autoencoder-GAN architecture to accurately recover missing values. TsImpute [29] distinguishes likely dropouts from true zeros using zero-inflated negative binomial (ZINB) modeling, followed by inverse distance weighted (IDW) clustering for final imputation. These methods synergize the strengths of diverse technologies, significantly enhancing imputation quality, though often at the expense of increased computational complexity. Additionally, SCRABBLE [13] integrates bulk RNA-seq data with single-cell RNA-seq data, while netSmooth (network Smoothing) [10] incorporates protein-protein interaction networks into single-cell RNA-seq analysis. Although the integration of multi-modal or multi-omics data offers an effective means to mitigate the limitations of individual datasets, it also presents substantial computational challenges, including data retrieval, alignment, and matching, which must be carefully addressed to ensure reliable results.

At the computational architecture level, existing methods can be divided into two categories: direct imputation and indirect imputation.

**(1) Direct Imputation**

It refers to approaches that uniformly estimate and fill all missing values in the expression matrix without distinguishing their biological or non-biological zeros. Among the methods discussed earlier, the following algorithms employ direct imputation: G2S3, scGNN, scTSSR, scBERT, GraphSCI, and scAMF. In Table 1, we denote these methods as *Y*.

**(2) Indirect imputation**

In indirect imputation methods, two distinct strategies are employed to address missing values in scRNA-seq data. The first strategy, explicit or implicit differentiation and imputation of non-biological zeros, relies on statistical or generative models to explicitly distinguish between biological and non-biological zeros during the imputation process. Methods like scRMD, scVI, SAVER/SAVERX, SCRABBLE, DCA, and bayNorm use probabilistic frameworks such as zero-inflated negative binomial (ZINB) distributions or Bayesian hierarchical models to parameterize technical noise and biological signals separately. Besides, implicit differentiation and imputation leverage model architectures to inherently separate noise from biological signals without explicitly labeling zero types. Techniques such as MAGIC, netSmooth, GEImpute, scRNMF, and scGGAN capture underlying biological patterns while filtering out technical noise. These models impute only the values identified as non-biological zeros while preserving biological zeros, ensuring that true biological silence is maintained. In Table 1, we denote these methods as *N*_1_.

The second strategy, two-stage predict-then-impute approaches, splits the imputation process into sequential steps. In the first stage, non-biological zeros are predicted using heuristic rules or statistical criteria, such as deviations from cluster-specific expression patterns (e.g., DrImpute, scImpute, SDImpute, VIPER, TsImpute, AGImpute, and SIMPLEs). Methods like ALRA and CMFImpute refine this by combining low-rank approximation with adaptive thresholding to preserve true zeros. In Table 1, we denote these methods as *N*_2_.

Current methods for imputing single-cell RNA sequencing data demonstrate diverse strategies to address technical noise and sparsity, yet they exhibit shared strengths and limitations. Approaches grounded in statistical models excel at distinguishing technical artifacts from biological signals through explicit assumptions, thereby preserving biological. However, their performance heavily relies on predefined distributional priors, which often fail to adapt to the complex, heterogeneous patterns observed in real-world datasets. Methods leveraging local data structures, such as cell or gene similarity networks, effectively maintain topological features and enhance tasks like visualization or trajectory inference. Nevertheless, their reliance on global smoothing operations risks obscuring cellular heterogeneity, which can particularly compromise particularly compromising sensitivity to rare cell populations or transitional states. Deep learning-based techniques, while powerful in capturing nonlinear, high-dimensional relationships and enabling cross-dataset generalization, suffer from limited interpretability due to their “black-box" nature, hindering applications in mechanistic biological studies. Indirect two-stage strategies, which decouple noise prediction from imputation, strike a balance between interpretability and controlled correction. However, their efficacy depends on the accuracy of initial noise detection, and computational scalability remains challenging for large-scale datasets.

Motivated by these advanced methods, we present D3Impute, a novel hybrid method that uniquely integrates dual embeddings of cell-cell and gene-gene networks within a low-dimensional space. (see [4]). In addition to biological zeros, other non-biological zero values encompass technical zeros and sampling zeros, with non-biological zeros being particularly challenging to predict. Thus, in this study, we posit that non-biological zeros essentially reflect the spatial expression patterns of genes in cells, which can be inferred through cellular and gene-gene relationships. Unlike existing approaches, D3Impute combines scRNA-seq and bulk RNA-seq data to precisely distinguish between biological and non-biological zeros before imputation, ensuring that only true dropout events are corrected. This novel zero-preserving mechanism significantly enhances the accuracy and reliability of downstream analyses. To validate its effectiveness, we evaluated D3Impute on multiple simulated and real scRNA-seq datasets, benchmarking it against state-of-the-art imputation methods. Our results demonstrate that D3Impute consistently outperforms these methods in key downstream analyses, such as differential expression analysis, cell clustering, and cell trajectory inference. These findings underscore D3Impute’s unique ability to not only improve the accuracy of scRNA-seq data imputation but also to preserve biological relevance, making it a valuable tool for advancing single-cell genomics research.

The organization of the article is as follows: Sect 2 describes the materials and the newly proposed algorithm D3Impute, Sect 3 describes the experiments and results, Sect 4 presents the discussions, and finally, Sect 5 summarizes the entire article.

## Materials and methods

### Dataset

To systematically evaluate the performance of our D3Impute algorithm, we established a comprehensive benchmarking framework incorporating six publicly available single-cell and bulk RNA sequencing datasets. As summarized in Table 2, all bulk RNA-seq datasets were rigorously paired with scRNA-seq counterparts from matching species (human) and tissue origins, with both data types required to meet stringent quality thresholds (>80% alignment rate). These datasets were specifically chosen based on their (i) biological diversity across human tissue types, (ii) established experimental validity, and (iii) widespread adoption as reference standards within the single-cell genomics community.

**Table 2:** Summary of datasets.

Data	Acession Code	Data Type	Tissue	Cell/Sample	Gene	Cell Type
Siletti [32]	-	scRNA-seq	Human Brain	4714	5935	11
Booth [33]	GSE120306	Bulk RNA-seq	Human Brain	7	19658	-
Guo2 [34]	GSE63818	scRNA-seq	Human Primordial Germ Cells	317	23787	2
Ito [35]	GSE167570	Bulk RNA-seq	Human Primordial Germ Cells	2	22866	-
Petropoulos [36]	-	scRNA-seq	Human Preimplantation Embryos	1529	21749	5
Voorden [37]	GSE250424	Bulk RNA-seq	Human Trophoblast Stem Cells	14	27449	-
iPSC [5]	-	scRNA-seq	Human iPS Cells	315	15724	5
Niwa [38]	GSE275240	Bulk RNA-seq	Human iPS Cells	18	40982	-
Pollen [39]	SRP041736	scRNA-seq	Human Cerebral Cortex	249	8869	11
Rao	GSE244006	Bulk RNA-seq	Human Cerebral Cortex	8	36164	-
CellType [40]	GSE75748	scRNA-seq	Human ES-derived Progenitors	1018	19097	7
CellType [40]	GSE75748	Bulk RNA-seq	Human ES-derived Progenitors	19	19097	7

These datasets encompass diverse biological contexts—from neurodevelopment to embryogenesis—providing a robust foundation for evaluating our method’s generalizability. The included datasets vary substantially in scale, ranging from smaller gene sets (∼5000 genes) to comprehensive transcriptome coverage (∼30000 genes), while containing between 2 to 11 distinct cell types, enabling thorough assessment of our method’s performance across different data complexities.

### D3Impute framework

**Fig 1:**
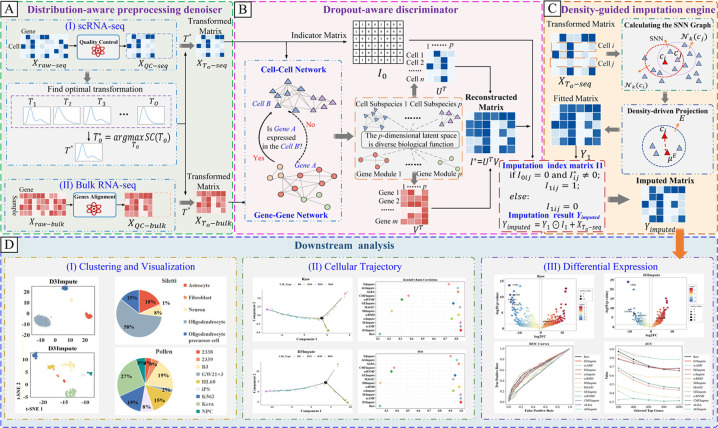
Workflow of D3Impute framework. (A) Distribution-aware preprocessing denoiser: Quality control and denoising of raw scRNA-seq (*X*_*raw*−*seq*_) and bulk RNA-seq (*X*_*raw*−*bulk*_) matrices. (B) Dropout-aware discriminator: Construction of interaction networks and generation of imputation index matrix *I*_1_. (C) Density-guided imputation engine: SNN graph-based neighbor identification and matrix updating $Y_{imputed}=Y_{1}⊙I_{1}+X_{T_{o}-seq}$. (D) Downstream analysis: Clustering, trajectory inference, and differential expression analysis.

As illustrated in Fig 1, the D3Impute framework consists of three synergistic computational modules: (i) a distribution-aware preprocessing denoiser, (ii) a dropout-aware discriminator, and (iii) a density-guided imputation engine.

- Distribution-aware preprocessing denoiser: This module performs adaptive normalization by modeling the statistical properties of gene expression distributions. It systematically reduces technical noise while preserving biologically meaningful variation across cell populations, establishing a mathematically grounded foundation for downstream analysis.
- Dropout-aware discriminator: This component employs a probabilistic model that leverages integrated cell–gene relationship modeling within a dual-network architecture. By combining cell-cell interaction networks from scRNA-seq data with gene co-expression networks derived from bulk RNA-seq, it accurately identifies non-biological zeros without the need for explicit distinction between technical artifacts and sampling limitations.
- Density-guided imputation engine: This engine utilizes a neighborhood-preserving strategy constrained by cellular manifold density. Through dynamic weight regulation in shared nearest neighbor (SNN) graphs, it selectively reconstructs dropout values identified by the discriminator. This approach conserves local heterogeneity and global data structure, thereby preventing over-smoothing and maintaining genuine cell-to-cell variation.

### Distribution-aware preprocessing denoiser

To ensure analytical fidelity, we implemented a multi-stage quality assurance protocol addressing both cellular and genomic integrity. The pipeline comprised two sequential filtering phases:

#### Quality control strategy.

(1) Cellular QC control

Cellular viability assessment integrated three orthogonal metrics: (i) Transcriptome completeness: Minimum 200 detectable genes in each cell; (ii) Sequencing saturation: Upper thresholds defined per dataset via nFeature_RNA distributions; (iii) Mitochondrial integrity: Sample-specific percent.mt cutoffs. The dataset-specific threshold optimization followed benchmarking studies, as shown in Table 3.

**Table 3:** Cellular QC parameters for each dataset.

Dataset	n Feature_RNA	percent.mt
Siletti	3000	1%
iPSC	4000	5%
Pollen	7500	5%
Guo2	8000	5%
CellTypes	10000	5%
Petropoulos	12000	5%

(2) Genomic QC control

Post-cellular filtration, we applied: (i) Ubiquity filter: Genes detected in <3 cells excluded; (ii) Feature selection: Identified top 2000 HVGs using Seurat’s variance stabilization.

The raw single-cell RNA-seq data structure was mathematically defined as $X_{raw-seq}\in {\mathbb{R}}^{N\times M}$, where *N* represents the initial cellular population and *M* denotes the complete genomic features. Following the quality control strategy, this matrix underwent dimensional reduction to $X_{QC-seq}\in {\mathbb{R}}^{n\times m}$, where *n* indicates the retained viable cell (*n* < *N* after Cellular QC filtration) and *m* corresponds to the selected highly variable genes (HVGs).

Concurrently, bulk RNA-seq data maintained a distinct matrix configuration $X_{raw-bulk}\in {\mathbb{R}}^{Z\times Q}$ (*Z* experimental samples in *Q* genomic features), where the feature space dimensionality *Q* substantially exceeded that of single-cell data (Q≫M). Intersection analysis between HVGs and bulk RNA-seq features was conducted to establish cross-platform integration. This process generated aligned expression matrices, designated as $X_{QC-seq}\in {\mathbb{R}}^{n\times m}$ for scRNA-seq and $X_{QC-bulk}\in {\mathbb{R}}^{Z\times m}$ for bulk RNA-seq, where m≤2000 represents the dimensionality of intersecting genes.

#### Transformation optimization.

To address the inherent heterogeneity in scRNA-seq data distributions, we developed a distribution-aware transformation framework. A candidate library comprising several mathematical methods was systematically evaluated to identify optimal preprocessing strategies. This approach specifically targets three technical challenges: (i) Normalizing scale disparities across experimental batches/cell types, (ii) Correcting skewed distributions in high-dimensional sparse data, and (iii) Enhancing signal-to-noise ratios for improved clustering sensitivity. We formalized the selection process through a performance metric by Eq (1).

$$T_{o}^{*}=\underset{T_{o}\in 𝛤}{\mathrm{arg max}}\;SC(T_{o}(X_{QC-*})),$$ (1)

where 𝛤 denotes the set of candidate transformations $\{T_{1},T_{1},\ldots \}$, *X*_*QC*−*_ denote the QC matrix (*∈{seq,bulk}), SC(·) denote silhouette coefficient evaluating clustering performance. $T_{o}^{*}$ denotes the optimal transformation maximizing cluster separation. Therefore, the transformed single-cell matrix is denoted by $X_{T_{o}-seq}=T_{o}^{*}(X_{QC-seq})\in {\mathbb{R}}^{n\times m}$, while the tranformed bulk matrix is denoted by $X_{T_{o}-bulk}=T_{o}^{*}(X_{QC-bulk})\in {\mathbb{R}}^{Z\times m}$.

### Dropout-aware discriminator

To establish a model for predicting whether genes are expressed in cells, we first constructed a cell interaction network and a gene interaction network based on preprocessed data. Additionally, to improve computational efficiency, we perform sparsification to predict the quantitative expression of genes in cells, specifically identifying the location of non-biological zero values. It is hypothesized that gene expression exhibits cell-type specificity, while the overall gene co-expression networks display module-specific characteristics due to functional differences among genes. To this end, we construct a cell-cell interaction network using single-cell transcriptomic sequencing data and a gene co-expression network using bulk transcriptomic data. By assuming these networks share a common low-dimensional embedding space, we predict the positions of non-biological zero values using the reconstructed low-rank matrix.

#### Establishment of sparse cell network and gene network.

In this part, we used scRNA-seq data to obtain a cell-cell interaction network and used bulk data to obtain a gene co-expression network. For the convenience of subsequent calculation, we use the k-nearest neighbor (k-NN) method to obtain the sparse cell weight network and the sparse gene weight network, respectively.

**Step 1. The indicator matrix is established for reconstruction.**

We obtained an indicator matrix denoted by *I*_0_ from the original scRNA-seq matrix $X_{T_{o}-seq}$, that is, the indicator matrix $I_{0}\in {\mathbb{R}}^{n\times m}$, and the element whose element is zero in the original matrix is also denoted by 0 in *I*_0_; Non-zero elements of the original matrix are denoted as 1 in *I*_0_. This indicator matrix is the prepared target that we need to reconstruct during the model process.

**Step 2. Cell-gene dual networks are established for prediction.**

We utilize scRNA-seq data and bulk gene expression data to calculate Pearson correlation coefficients row-wise and column-wise, respectively, thereby obtaining cell-cell and gene-gene similarity matrices $R^{c}\in {\mathbb{R}}^{n\times n}$ and $R^{g}\in {\mathbb{R}}^{m\times m}$.

**Step 3. Sparse matrices are established for simple computation.**

We employ the k-NN sparsification method to sparsify the similarity matrices *R^c^* and *R^g^*. Based on the original $X_{T_{o}-seq}$ matrix, for each row (i.e., for every pair of the cell *c*_*i*_ and the cell *c*_*j*_), we sort them according to the similarity measure in *R^c^*. We define *S*_*ij*_ as:

$$S_{ij}=\left\{ \begin{array}{cc} 1, & if\;c_{j}\in {𝒩}_{k}(c_{i})\;and\;c_{i}\in {𝒩}_{k}(c_{j})\\ 0, & if\;c_{j}\notin {𝒩}_{k}(c_{i})\;and\;c_{i}\notin {𝒩}_{k}(c_{j})\\ 0.5, & otherwise \end{array}\right.,$$ (2)

where ${𝒩}_{k}(c_{i})$ denotes the k-nearest neighbors for cell *c*_*i*_. Then, we obtain the sparsified representation of the similarity matrix *R^c^* by computing the element-wise (Hadamard) product via Eq (3):

$$R^{c*}=S⊙R^{c}.$$ (3)

The same operation is applied to *R^g^*, resulting in the sparsified matrix R^g^*. Thus, sparse cell network R^c^* and sparse gene network R^g^* are prepared for reconstructing the indicator matrix *I*_0_.

#### Graph-regularized latent space projection.

We propose a joint embedding framework that projects both gene and cell networks into a shared low-dimensional latent space. Let $V\in {\mathbb{R}}^{p\times m}$ and $U\in {\mathbb{R}}^{p\times n}$ denote the latent feature matrices for genes and cells, respectively, where *p* represents the reduced dimensionality (p≪n,m). Given an initial indicator matrix $I_{0}\in {\mathbb{R}}^{n\times m}$, we optimize the latent representations to satisfy $I_{0}\approx U^{T}V$ while incorporating three key constrains.

(1) Reconstruction accuracy:

$$E_{recon}=\|I_{0}-U^{T}V{\|}_{F}^{2}.$$ (4)

This term ensures the low-rank approximation $U^{T}V$ faithfully represents the original data matrix *I*_0_. To preserve biological interpretability and maintain consistency with the non-negative nature of scRNA-seq data, we impose non-negativity constraints on both factor matrices, i.e., U≥0 and V≥0.

(2) Tikhonov regularization:

$$E_{tikhonov}=\beta (\|U{\|}_{F}^{2}+\|V{\|}_{F}^{2}).$$ (5)

The *L*_2_-norm constraints prevent overfitting by controlling the magnitude of latent features.

(3) Dual graph regularization:

$$E_{graph}=\lambda_{c}Tr(UL_{c}U^{T})+\lambda_{g}Tr(VL_{g}V^{T}).$$ (6)

The graph Laplacian matrices *L*_*c*_ (cell graph Laplacian) and *L*_*g*_ (gene graph Laplacian) are constructed from the sparsified similarity matrices *R^c^* and R^g^*, respectively. Specifically, the degree matrix *D*_*c*_ is a diagonal matrix where each diagonal element $(D_{c}{)}_{ii}$ is the sum of the *i*-th row of R^c^*, i.e., $(D_{c}{)}_{ii}=\sum_{j}(R^{c*}{)}_{ij}$ representing the total connection weight of cell *i* in the network. The gene degree matrix *D*_*g*_ is computed analogously from R^g^*. The graph Laplacian is then defined as $L_{c}=D_{c}-R^{c*}$ (and similarly for *L*_*g*_), which encodes the topological structure of the respective networks. The final complete objective function combines these components:

$$min(U,V)\;E_{recon}+E_{tikhonov}+E_{graph}\;\text{subject to}\;U\geq 0,\;V\geq 0.$$ (7)

Based on the Karush–Kuhn–Tucker (KKT) criterion [41], *u*_*li*_ and $v_{li}$ were obtained:

$$u_{li}\leftarrow u_{li}\frac{(VI_{0}^{T}+\lambda_{c}UR^{c*}{)}_{li}}{(VV^{T}U+\beta U+\lambda_{c}UD_{c}{)}_{li}}\;v_{li}\leftarrow v_{li}\frac{(UI_{0}^{T}+\lambda_{g}VR^{g*}{)}_{li}}{(UU^{T}V+\beta V+\lambda_{g}VD_{g}{)}_{li}}.$$ (8)

Update matrices *U* and *V* until convergence or the upper limit of the number of iterations is reached. Afterward, $U^{T}V$ is computed to obtain the reconstructed matrix $I^{*}\in {\mathbb{R}}^{n\times m}$. Detailed derivation of the update rules and convergence analysis are provided in Supporting information S1 Text.

#### Non-biological zero value identification.

This way, through comparing the I^*^ and *I*_0_ matrices, we can derive an imputation index matrix *I*_1_ containing biological zeros. Whenever an element is 0 in *I*_0_ but non-zero in I^*^, the corresponding position in *I*_1_ will be assigned 1, with all other elements set to 0. The 1’s indicate the predicted gene expression positions in corresponding cells, which need imputation, while the remaining 0’s are true biological zeros that should be preserved. Having obtained *I*_1_, we now know precisely which positions require imputation.

### Density-guided imputation engine

To determine imputation values, we leverage the biological principle that spatially or transcriptionally proximate cells exhibit similar gene expression profiles. Our approach employs geometric mapping to compute a neighborhood-weighted average, reconstructing an n×m single-cell expression matrix *Y*_1_ through the following steps. Here, samples represent individual cellular data units, formally defined as a cell set $C=\{c_{1},c_{2},...,c_{n}\}$, where *n* is the total number of cells.

**Step 1. Identification of Top Shared Nearest Neighbors.**

For each target cell *c*_*i*_, we identify its top *k* neighbors based on shared nearest neighbors (SNNs). Specifically, for every $c_{j}\in {𝒩}_{k}(c_{i})$, we compute the SNN set:

$$\text{SNN}(c_{j},c_{j})={𝒩}_{k}(c_{i})\cap {𝒩}_{k}(c_{j}).$$ (9)

Cells $c_{j}\in {𝒩}_{k}(c_{i})$ are then ranked in descending order of $\left|\text{SNN}(c_{i},c_{j})\right|$, the top *k* cells with the highest SNN overlap form the set *E*:


$$E=\{c_{\left(1\right)},c_{\left(2\right)},\ldots ,c_{\left(k\right)}\}.$$

Using *E*, we estimate the mapping centroid *F*(*c*_*i*_) as the mean expression of cells in *E* by Eq (10).

$$F(c_{i})=\frac{1}{\left|E\right|}\sum_{c_{j}\in E}c_{j}.$$ (10)

**Step 2. Optimal Projection for Imputation.**

We define candidate projections along the direction $F(c_{i})-c_{i}$ using a set of weight values $t\in \left\{t_{1},t_{2},\cdots \right\}$:

$$c_{t}=c_{i}+t(F(c_{i})-c_{i}).$$ (11)

The optimal projection $c_{t}^{*}$ is selected by minimizing the sum of squared distances to refined neighborhoods *E*, equivalent to maximizing the local density metric $\rho (c_{t})$:

$$\rho (c_{t})=\frac{1}{\sum_{c_{t}\in E}{\left\|c_{i}-c_{t}\right\|}_{2}^{2}}.$$ (12)

The final imputed value $c_{t}^{*}$ corresponds to the projection with the highest density and smallest distance sum

$$c_{t}^{*}=\underset{c_{t}}{argmax}\;\rho (c_{t}).$$ (13)

During this process, the original gene expression matrix $X_{T_{o}-seq}$ is iteratively updated to *Y*_1_. The matrix *Y*_1_ preserves the structural information and heterogeneity of the original data. The complete imputation results are obtained through element-wise multiplication with the indicator matrix and the original matrix $X_{T_{o}-seq}$ by Eq (14)

$$Y_{imputed}=Y_{1}⊙I_{1}+X_{T_{o}-seq}.$$ (14)

## Results

To rigorously benchmark our method, we performed systematic comparisons against 12 state-of-the-art single-cell imputation approaches (shown in Table 1, which are marked with horizontal lines). The selected methods span state-of-the-art imputation approaches from 2018 to 2024, carefully chosen to represent distinct methodological categories—including established classical techniques and emerging novel frameworks—based on their prevalence in current practice and technical innovation. All reference methods were implemented strictly following their original publications’ specifications, using default parameters unless otherwise noted.

Our results begin with an examination of the model’s parameter settings and controlled simulations on masked data. We then detail the optimization approaches, followed by an in-depth exploration of each computational module’s biological significance and experimental validation. We conclude by showcasing the method’s performance across three fundamental downstream applications—cell type clustering, pseudotime reconstruction, and differential expression analysis—providing comprehensive evidence of its practical utility in single-cell data analysis. Finally, we report on the computational efficiency of the proposed framework. All experiments were executed on a Windows 10 platform (Intel Core i7 @5.20GHz, 32GB RAM) using MATLAB R2022b and R 4.4.1 environments.

### Compared algorithms

We conducted a systematic comparative analysis of 12 state-of-the-art scRNA-seq imputation methods, evaluating them across two critical dimensions: (i) methodological categorization and (ii) zero-inflation handling capability (direct vs. indirect imputation approaches). Our comprehensive benchmarking framework incorporates detailed specifications for each method, including algorithmic implementations (with source code availability), programming language dependencies, methodological classification, and imputation strategy types, all meticulously documented in Table 1.

### Evaluation measures

In clustering analysis, true labels represent the ground-truth class assignments of samples, denoted as $X=\{x_{1},x_{2},...,x_{n}\}$, where $x_{i}\in \{1,2,...,K\}$ indicates the true class of sample *c*_*i*_ (with *K* classes in total). Predicted labels are the cluster assignments generated by a clustering algorithm, denoted as $Y=\{y_{1},y_{2},...,y_{n}\}$, where $y_{i}\in \{1,2,...,L\}$ represents the cluster assigned to sample *c*_*i*_ (with *L* clusters in total). The evaluation metrics categorized by their specific applications in our study are presented below.

**Skewness coefficient (SK)**: quantifies the asymmetry in gene expression distributions, guiding the selection of appropriate data transformation methods. It measures the deviation from a normal distribution by Eq (15)

$$\text{SK}=\frac{n}{\left(n-1)(n-2\right)}\sum_{i=1}^{n}(\frac{c_{i}-\bar{c}}{\sigma }{)}^{3},$$ (15)

where $\bar{c}$ is the sample mean, and *σ* is the sample standard deviation. SK>0 indicate right-skewed distribution; SK=0 indicate symmetric distribution; SK<0 indicate left-skewed distribution.

**Silhouette coefficient (SC):** Evaluates clustering quality based on intra-cluster compactness and inter-cluster separation, without requiring true labels *X*. For a sample *c*_*i*_ with the predicted label *y*_*i*_ and the cell set C_*_ for the *-th class, let $A(c_{i})=\frac{1}{|C_{y_{i}}|-1}\sum_{c_{i},c_{j}\in C_{y_{i}},j\neq i}d(c_{i},c_{j})$ measure its average distance to other samples in the same cluster (intra-cluster compactness) and $B(c_{i})=min_{C_{k}\neq C_{y_{i}}}\frac{1}{\left|C_{k}\right|}\sum_{c_{j}\in C_{k}}d(c_{i},c_{j})$ the smallest average distance to sample in other clusters (inter-cluster separation). The per-sample SC is


$$\text{SC}(c_{i})=\frac{B(c_{i})-A(c_{i})}{max\{A(c_{i}),B(c_{i})\}},$$

and the global SC is the average across all samples defined by Eq (16)

$$SC=\frac{1}{n}\sum_{i=1}^{n}\text{SC}(c_{i}).$$ (16)

It ranges from –1 to 1, where higher values indicate better clustering performance.

**Normalized mutual information (NMI):** Measures information overlap between predicted single-cell RNA-seq clustering against annotated cell types using entropy.

$$\text{NMI}=\frac{2I(X,Y)}{H(X)+H(Y)},$$ (17)

It ranges from 0 to 1, where 1 signifies perfect alignment (all information shared) and 0 indicates no dependency between *X* and *Y*.

**Ajusted rand index (ARI):** Evaluates the agreement between true labels *X* and predicted labels *Y* by comparing the consistency of pairwise sample assignments. Let *a* be the number of cell pairs correctly assigned to the same cluster by a clustering method. *b* is the number of cell pairs incorrectly assigned to the same cluster. *c* is the number of cell pairs incorrectly assigned to different clusters, and *d* is the number of cell pairs correctly assigned to different clusters. The ARI is calculated by Eq (18):

$$\text{ARI}=\frac{2(ad-bc)}{\left(a+b)(b+d)+(a+c)(c+d\right)},$$ (18)

which ranges from –1 to 1. ARI=1 indicates perfect match; ARI=0 indicates random-level agreement; ARI=−1 indicates systematic discordance.

**Pseudo-temporal ordering score (POS) [42]:** Validates whether inferred cell differentiation trajectories align with known biological developmental timelines.

$$\text{POS}=\sum_{i=1}^{n}\sum_{j:j>i}g(\pi ,c_{i},c_{j}),$$ (19)

where *π* represents an ordered path of *n* cells generated by a specific trajectory inference method. Let $g(\pi ,c_{i},c_{j})$ be the score that describes how well the order of cell *c*_*i*_ and cell *c*_*j*_ in the ordered path *π* matches the external order label. POS is defined as the sum of all cell pairs. It ranges from –1 to 1. POS=1 indicates algorithm perfect reconstruction trajectories; POS=0 indicates pseudotime independent of true stages; POS=−1 indicates inverse ordering.

**Kendall’s rank correlation (Kendall) [43]:** Assesses the rank concordance of cells along the pseudo-time axis against their true developmental order.

$$\text{Kendall}=\frac{2(J-W)}{n(n-1)},$$ (20)

where *J* and *W* denote the number of concordant and discordant cell pairs, respectively. Two pairs of observations are said to be concordant when their magnitude relationship agrees with the reference relationship. It ranges from –1 to 1; Kendall=1 indicates perfect agreement in rankings; Kendall=0 indicates random ordering; Kendall=−1 indicates perfect inverse ranking.

**Receiver operating characteristic (ROC) curve:** Evaluates the performance by comparing imputed/observed data-derived differentially expressed genes (DEGs) with a reference set at varying statistical thresholds.

$$\text{TPR}=\frac{\text{TP}}{\text{TP}+\text{FN}},\;\text{FPR}=\frac{\text{FP}}{\text{FP}+\text{PN}},$$ (21)

where TPR means true positive rate and FPR means false positive rate. ROC curve plots TPR (y-axis) against FPR (x-axis). The Area Under the ROC Curve is the metric AUC.

### Parameter settings

D3Impute incorporates five key parameters that require careful calibration, including the sparseness constraint coefficient *β*, graph regularization coefficients $\lambda_{c}$ and $\lambda_{g}$ for cells and genes, respectively, latent subspace dimensionality *p*, and neighborhood size *k*. To ensure the robustness and generalization capability of D3Impute across diverse datasets, we systematically analyzed the influence of its five key parameters and implemented a multi-stage grid search strategy for parameter optimization.

The sparsity constraint coefficient *β* regulates the Frobenius norm regularization terms $\|U{\|}_{F}^{2}\;+\;\|V{\|}_{F}^{2}$ applied to the latent feature matrices, serving as a mechanism to control model complexity and mitigate overfitting. Lower values (e.g., 0.0001) permit greater flexibility in reconstructing the input matrix *I*_0_ but may capture noise, whereas higher values (e.g., 0.1) enforce stronger regularization, enhancing numerical stability at the potential expense of expression recovery fidelity. We search from 0.0001 to 0.1 with a step size of 0.0005.

The graph regularization parameters $\lambda_{c}$ and $\lambda_{g}$ modulate the influence of cellular and genomic topological structures through the Laplacian regularization terms $Tr(UL_{c}U^{T})$ and $Tr(VL_{g}V^{T})$, respectively. These terms enforce smoothness constraints on the latent embeddings *U* and *V* relative to the constructed cell and gene graphs. Diminished values may lead to insufficient preservation of manifold structures, while elevated values could overly constrain the solution space, potentially obscuring biologically relevant expression patterns. We set $\lambda_{c}=\lambda_{g}$ and searched from 0.0001 to 0.1 with a step size of 0.0005 as well.

The neighborhood parameter *k* determines the sparsity of the *k*-nearest neighbor graphs underlying the affinity matrices R^c^* and R^g^*, which subsequently define the graph Laplacians *L*_*c*_ and *L*_*g*_. Reduced *k* values yield sparser graphs that emphasize local connectivity, suitable for capturing fine-grained cellular and genomic heterogeneity, whereas increased *k* values produce denser graphs that promote global consistency but may introduce spurious connections. Based on the sample size *n*, we searched from ${\text{log}}_{2}^{n}$ to $\sqrt{n}$ with a step size of 2.

The subspace dimension *p* controls the expressiveness of the embedding space. Smaller values (e.g., 10) aid in denoising and enhance cluster clarity but may lose biological details, while larger values (e.g., 100) improve expressiveness but increase the risk of overfitting. We searched from 10 to 100 with a step size of 5.

We employed a three-stage grid search approach for parameter optimization:

- Stage I: Jointly search *β* and *λ* ($\lambda =\lambda_{c}=\lambda_{g}$) to determine the optimal regularization parameter combination, aiming to maximize clustering performance (Adjusted Rand Index, ARI) and visualization separation (Silhouette coefficient, SC).
- Stage II: With *β* and *λ* fixed, search for the neighbor number *k* to optimize the locality of graph structutre construction.
- Stage III: With the first three parameters fixed, search for subspace dimension *p* to balance expressiveness and model stability.

**Fig 2:**
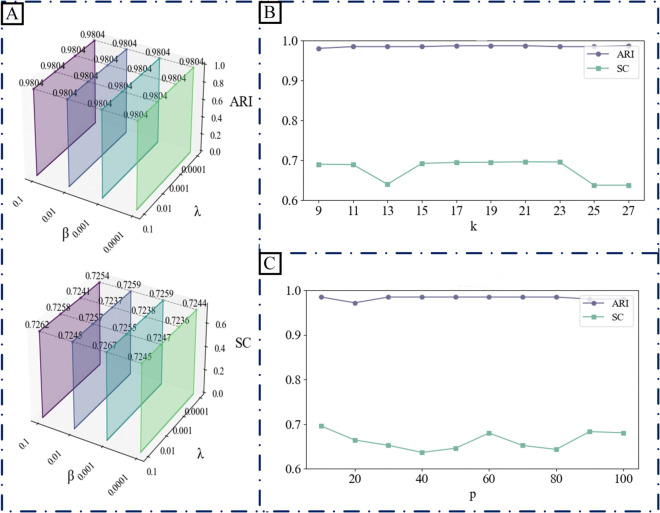
Parameter optimization analysis for D3Impute on the Siletti dataset. (A) A waterfall plot evaluates sixteen parameter combinations of *β* and *λ* (where $\lambda =\lambda_{c}=\lambda_{g}$), revealing optimal model performance at β=0.001 and λ=0.1. (B) Further refinement yielded the best-performing hyperparameter at *k* = 23. (C) Further refinement yielded the best-performing hyperparameter at *p* = 10.

The parameter optimization process for the Siletti dataset, as illustrated in Fig 2, revealed that optimal regularization was achieved at β=0.001 and λ=0.1 (with $\lambda_{c}=\lambda_{g}=\lambda$). Model performance showed a clear inverse relationship with subspace dimensionality *p*, with optimal results consistently obtained at *p* = 10 across all datasets. Subsequent optimization yielded *k* = 23 as the ideal value for the Siletti dataset. The complete parameter configurations across five datasets are presented in Table 4, demonstrating several important patterns in parameter selection. The subspace dimensionality *p* remained consistently at 10 for all datasets, while regularization parameters showed greater variability that reflected dataset-specific characteristics. Neighborhood size *k* exhibited substantial variation (range: 6-23), correlating with dataset complexity. Additional parameter optimization results for other datasets are presented in Supporting information S1 Fig–S5 Fig.

**Table 4:** Parameter setting for six datasets.

Parameter	Siletti	Guo2	Pollen	iPSC	Petropoulos	CellType
β	0.001	0.0001	0.1	0.1	0.01	0.01
$\lambda_{c}$	0.1	0.0001	0.1	0.1	0.01	0.01
$\lambda_{g}$	0.1	0.0001	0.1	0.1	0.01	0.01
*k*	23	16	7	7	10	6
*p*	10	10	10	10	10	10

### Evaluation on simulated masking experiments

To quantitatively evaluate the imputation accuracy and robustness of D3Impute, where the ground truth is known, we conducted masking experiments on simulated data. Following the masking strategy of CMFImpute [16], we randomly masked non-zero values and assessed the accuracy of the imputed values against the original values using Pearson Correlation Coefficient (PCC) and Root Mean Square Error (RMSE), as implemented in scMOO [44].

We generated simulated datasets using Splatter, each comprising 2000 genes and 500 cells across three cell types, with a baseline zero rate of 26.83%. To mimic the dropout effects typical of real data scRNA-seq data, we applied three different dropout rates (60%, 70%, and 80%). For each dropout rate, we conducted five independent random masking trials using random seeds 44, 55, 66, 77, and 88, resulting in a total of 15 perturbed datasets. The actual zero proportions and skewness coefficients of the expression distribution for each dataset are provided in Table 5. Given the consistent skewness and expression distribution patterns observed across all datasets, we employed the same algorithm parameters (β=λ=0.01, *k* = 21, and *p* = 10) and transformation method (Log10) in all experiments to ensure comparability and stability of the evaluation.

**Table 5:** Summary of zero rate and skewness across five random seeds under varying dropout rates.

Dropout Rate	Zero Rate					Skewness				
	Seed 44	Seed 55	Seed 66	Seed 77	Seed 88	Seed 44	Seed 55	Seed 66	Seed 77	Seed 88
60%	70.66%	70.74%	70.70%	70.75%	70.67%	0.5013	0.5741	0.6952	0.7459	1.0367
70%	78.01%	78.03%	78.01%	78.05%	77.99%	0.5636	0.4935	0.6709	0.8148	0.9688
80%	85.33%	85.35%	85.34%	85.35%	85.32%	0.8653	0.6474	0.6027	0.8812	1.2519

**Fig 3:**
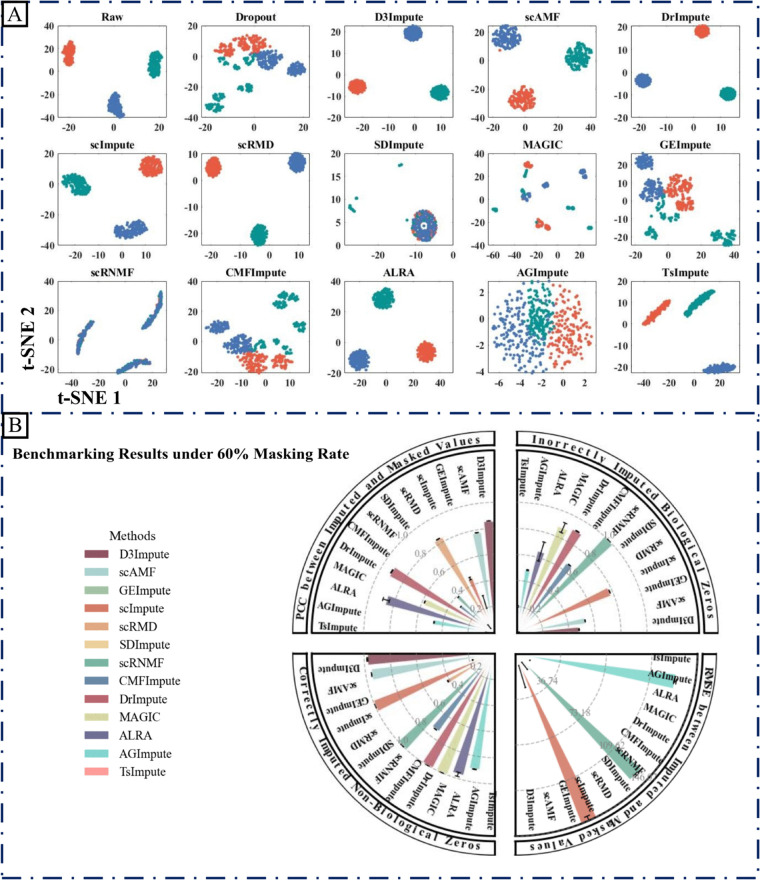
Benchmarking results under a 60% dropout rate. (A) 2D visualization of the masked dataset (seed = 55) before and after imputation, illustrating the restoration of data structure. (B) Circular quadrant plot summarizing the performance of multiple imputation algorithms across five random masking trials (seeds = 44, 55, 66, 77, 88). The circle is divided into four quadrants, each representing one evaluation metric: Pearson correlation coefficient (PCC), root mean square error (RMSE), proportion of correctly recovered non-biological zeros, and proportion of falsely imputed biological zeros. Within each quadrant, the bars show the mean performance of each algorithm over five random seeds, and the error bars indicate the corresponding standard deviation (mean ± SD).

The imputation results under the three dropout scenarios are presented in Fig 3, Supporting information S6 Fig and S7 Fig. Specifically, subfigures (A) in each of these figures display 2D visualizations of the data before and after imputation, where a gradual degradation in image quality is observed as the dropout rate increases. The four quantitative accuracy metrics—PCC, RMSE, proportion of correctly identified non-biological zeros, and proportion of falsely imputed biological zeros—are jointly visualized in the circular plot of Fig 3B for the 60% dropout rate. Corresponding metrics for dropout rates of 70% and 80% are shown in Supporting information S6 FigB–D and S7 FigB–D, respectively.

A comprehensive comparison demonstrates that the imputed results from D3Impute exhibit excellent agreement with the complete (Raw) data, reflected by higher PCC and lower RMSE values. Moreover, D3Impute performs well in zero-value identification: it accurately recognizes non-biological zeros (high identification rate) while effectively avoiding false imputation of biological zeros (low false positive rate). These outcomes underscore the methods’ ability to balance biological plausibility with technical accuracy.

### Module-wise effectiveness analysis

The D3Impute framework comprises three core computational modules: (i) a distribution-aware preprocessing denoiser, (ii) a dropout-aware discriminator, and (iii) a density-guided imputation engine. In this section, we present detailed architectural designs for each module and systematically evaluate their effectiveness through comprehensive comparative studies and ablation experiments.

#### Performance evaluation of distribution-aware denoiser.

To establish an optimal preprocessing strategy, we systematically evaluated seven distinct transformation methods across six scRNA-seq datasets. Our analysis, as summarized in Table 6, first reveals that the choice of data transformation profoundly influences downstream clustering outcomes. This finding underscores the limitation of a one-size-fits-all approach and highlights the necessity of a distribution-aware preprocessing step.

**Table 6:** Adjusted rand index (ARI) comparisons for seven transformation methods on six scRNA-seq datasets after D3Impute interpolation.

Method	Siletti	Guo2	Pollen	iPSC	Petropoulos	CellType
Value-to-rank	0.7593	0.9500	0.7917	0.5611	0.3052	0.587
Unit-vector	0.6627	0.8844	**0.9454**	0.6324	**0.5399**	0.5807
Log-normalization	0.6684	0.9680	0.6716	0.6306	0.2967	0.3697
Log2	0.9085	0.9980	0.4963	0.6301	0.3120	0.5287
Log10	0.9627	**0.9980**	0.5347	**0.6635**	0.3608	**0.7839**
Loge	0.9485	0.9980	0.4649	0.6068	0.3222	0.6147
Box-Cox	**0.9848**	0.5903	0.5885	0.4063	0.2907	0.4132

This observation forms the foundation of our distribution-aware denoiser module. The module implements a tailored strategy by selecting, for each dataset, the transformation that maximizes the Silhouette Coefficient (SC). The comprehensive SC metrics for all transformation-dataset combinations are detailed in Table 7, which explicitly highlights the module’s optimal choice for each dataset. The effectiveness of this data-specific denoising step is quantitatively demonstrated in the final column of Table 8, showing consistent and significant enhancement in clustering performance across all datasets, with a mean SC improvement of 9.18% (ranging from 1.18% to 28.70%). Notably, the substantial variation in optimal transformations across datasets indicates that data with different underlying distributions respond disparately to preprocessing methods, further validating the need for a customized denoising strategy.

**Table 7:** Silhouette coefficient (SC) comparisons for seven transformation methods on six scRNA-seq datasets.

Method	Siletti	Guo2	Pollen	iPSC	Petropoulos	CellType
Raw	0.5249	0.2322	0.0150	0.0954	0.3233	0.2109
Value-to-rank	0.0313	0.3038	0.1671	0.0141	0.1266	0.0825
Unit-vector	0.4152	0.3352	**0.3020**	0.1163	**0.3523**	0.1786
Log-normalization	0.2577	0.2847	0.2346	0.0226	0.1605	0.1021
Log2	0.4775	0.3020	0.1913	0.1577	0.2300	0.1395
Log10	0.5138	**0.3430**	0.1739	**0.1577**	0.1137	**0.2608**
Loge	0.5138	0.3430	0.1739	0.1577	0.1137	0.1198
Box-Cox	**0.5367**	0.3024	0.0872	0.0413	0.1834	0.1102

**Table 8:** Distributional statistics on six scRNA-seq datasets.

Dataset	Distribution type	Skewness	Transformation	Rationale
Siletti	Bimodal distribution	1.1→0.4	Box-Cox	SC↑1.18%
Guo2	Mild skewness	2.3→0.4	Log10	SC↑11.08%
iPSC	Mild skewness	1.5→0.2	Log10	SC↑6.23%
CellType	Mild skewness	0.6→0.1	Log10	SC↑4.99%
Pollen	Severe skewness	2.5→0.4	Unit-vector	SC↑28.70%
Petropoulos	Severe skewness	4.4→0.4	Unit-vector	SC↑2.90%

#### Performance evaluation of dropout-aware discriminator.

Our proposed D3Impute framework follows a hybrid, two-stage imputation strategy. This process involves: first, leveraging gene co-expression networks and cell interaction networks to project the data into a shared low-dimensional embedding space, within which we predict whether a zero count represents a true biological signal or a technical artifact. It is critical to clarify that the zeros in scRNA-seq data are a mixture of two distinct types: true biological zeros, which indicate the genuine absence of gene expression, and non-biological zeros, which are primarily caused by technical artifacts. Seminal work in the field has established that a dominant fraction of these non-biological zeros is attributable to dropout events, arising from the stochastic failure in capturing or amplifying low-abundance mRNA transcripts [45,46]. Given that dropout constitutes the major source of non-biological zeros, the terms “dropout" and “non-biological zero" are often used interchangeably in computational literature [9], a convention we adopt hereafter for simplicity. Therefore, our method is specifically designed to first identify the positions of these non-biological zeros/dropouts computationally, and then perform targeted imputation. In this section, we validate the effectiveness of this dropout-aware discriminator module through comparative experiments and biological significance analysis. Our designed imputation method belongs to a hybrid, two-stage data integration approach. The two-stage process is primarily reflected in: first, we model gene co-expression networks and cell interaction networks using two distinct types of data, using a shared low-dimensional embedding space as the medium to predict whether genes are expressed in cells. Therefore, our method is a missing-value identification approach that first predicts the positions requiring imputation through computational methods, then determines which values to impute to fill missing data. In this section, we will validate the effectiveness of the dropout-aware discriminator module through multiple comparative experiments and biological significance analyses.

We began the evaluation by comparing our discriminator against several indirect two-stage methods to assess its standalone performance. We then conducted systematic benchmarking with representative direct imputation approaches to assess the overall methodological advantage. Finally, we explored the biological relevance of the low-dimensional embedding space through functional enrichment analysis.

**(1) Comparative benchmarking against indirect two-stage imputation methods**

**Fig 4:**
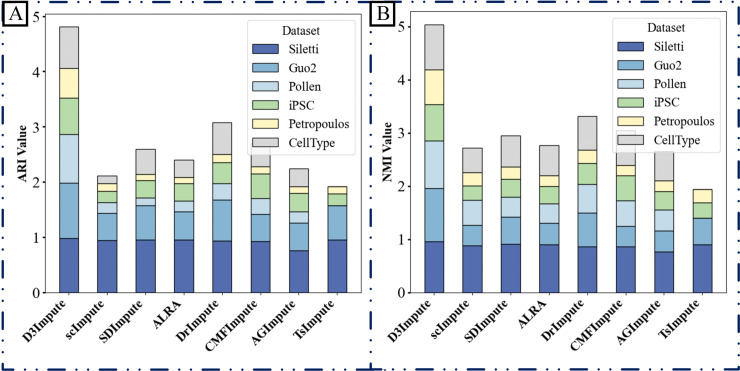
Clustering performance of imputed data using eight discrimination modules. A uniform imputation engine was applied across eight two-stage methods to assess clustering accuracy. The bar plots display (A) Adjusted Rand Index (ARI) and (B) Normalized Mutual Information (NMI) scores achieved across six datasets by integrating each method’s dropout-discrimination component (scImpute, SDImpute, ALRA, DrImpute, CMFImpute, AGImpute, TsImpute) with our density-guided imputation module.

To validate the performance of our dropout-aware discriminator module, we conducted comparative analyses with several two-stage methods (including scImpute, SDImpute, ALIRA, DrImpute, CMFImpute, AGImpute, and TsImpute) that employ a similar architectural design framework. While preserving their respective zero-discrimination procedures, we replaced their subsequent imputation steps with our density-guided imputation engine. The clustering performance of the resulting imputed data was quantitatively evaluated using the Adjusted Rand Index (ARI) and Normalized Mutual Information (NMI) metrics, as shown in Fig 4.

As illustrated in Fig 4, D3Impute’s dropout discriminator demonstrated superior performance compared to conventional indirect approaches in all benchmark datasets. This performance advantage stems from the framework’s innovative incorporation of cross-platform topological constraints, which effectively integrates complementary information from both single-cell (scRNA-seq) and bulk RNA-seq data. The consistent outperformance across diverse datasets provides compelling validation of our core hypothesis that gene co-expression networks derived from bulk RNA-seq offer essential contextual information for distinguishing technical artifacts from true biological signals in single-cell data.

**(2) Comparative benchmarking against direct imputation methods**

**Fig 5:**
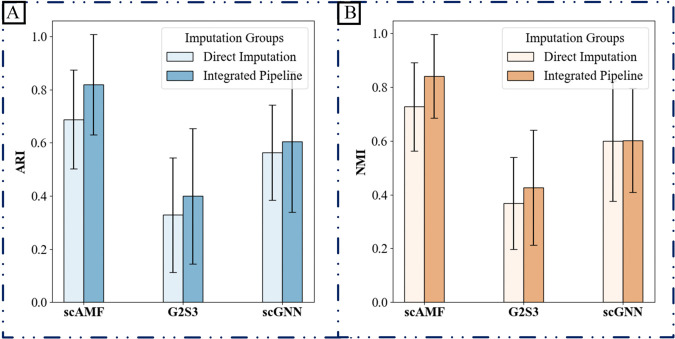
Performance enhancement of direct imputation methods by integrating the dropout-aware discriminator. The bar graphs compare the clustering performance, measured by (A) Adjusted Rand Index (ARI) and (B) Normalized Mutual Information (NMI), of standalone direct imputation methods (scAMF, G2S3, scGNN) against their versions integrated with our discriminator. Results are aggregated across six datasets, with detailed numerical results for each dataset available in Supporting information S1 Table and S2 Table.

To further assess the utility of our dropout-aware discriminator, we constructed hybrid pipelines by integrating it with several established direct imputation methods (scAMF, G2S3, and scGNN). In this integrated workflow, our discriminator was first applied to predict dropout events, after which the respective direct imputation method was used to fill only the predicted non-biological zeros. Comparative experiments demonstrated that this integrated strategy substantially enhanced clustering performance across all six benchmark datasets, as measured by ARI and NMI, while also better preserving biological variance. The consistent improvements observed confirm that our discriminator effectively complements direct imputation methods by providing a more accurate targeting of technical zeros prior to imputation. These results are summarized in Fig 5.

#### Performance evaluation of density-guided imputation engine.

We conducted a comparative evaluation between two imputation paradigms: (i) direct mean smoothing within neighborhoods and (ii) our proposed density-guided local projection.

The two methods exhibited comparable performance in regions of uniform cell distribution. However, pronounced differences emerged under suboptimal conditions, particularly during cell position updates guided by domain information. In these updates, cells typically migrate toward their respective cluster centroids along directional vectors (Fig 15E). While both methods performed similarly in standard scenarios, critical limitations of mean smoothing were exposed when a query cell *c*_*q*_ was erroneously assigned to the domain of cell *c*_*z*_. In such cases of misclassification, mean smoothing introduced substantial error, pulling *c*_*z*_ along an incorrect trajectory (Fig 15F, red arrows). In contrast, the density-guided approach effectively mitigated this artifact by identifying local maxima in data density through weighted projections, thereby correctly recentering *c*_*z*_.

**Fig 6:**
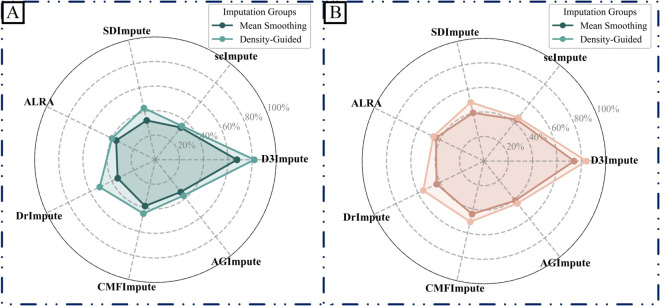
Comparison of two imputation paradigms on non-biological zeros. Density-guided and mean-smoothing strategies were applied to dropout values identified by different indirect algorithms. Radar plots illustrate the average performance of each imputation method across six benchmark scRNA-seq datasets. (A) Performance comparison of mean ARI across six datasets after applying each imputation method for non-biological zero correction. (B) Performance comparison of mean NMI across six datasets after applying each imputation method for non-biological zero correction. Detailed numerical results for all six datasets are provided in Supporting information S3 Table and S4 Table.

To further validate the general utility of our method, we implemented both imputation strategies—mean smoothing versus density-guided projection—within several leading frameworks, including D3Impute, scImpute, SDImpute, ALRA, DrImpute, CMFImpute, AGImpute, and TsImpute. As summarized in Fig 6, the density-guided imputation engine consistently achieved superior performance across evaluated metrics. Notably, while TsImpute was reported to perform well on the Pollen and CellType datasets in its original publication, we observed non-convergence issues when applying our standardized preprocessing pipeline, which incorporates rigorous quality control and filtering steps. Consequently, TsImpute was excluded from subsequent comparative analyses on these datasets.

#### Ablation studies.

To systematically evaluate the contribution of each component in D3Impute, we conducted a series of ablation experiments. The framework integrates three core modules: a distribution-aware data Transformation module (T), a dropout-aware Discrimination module (D), and a density-guided Imputation module (I). We compared four configurations: T only, T+I, D+I, and the complete T+D+I pipeline.

**Fig 7:**
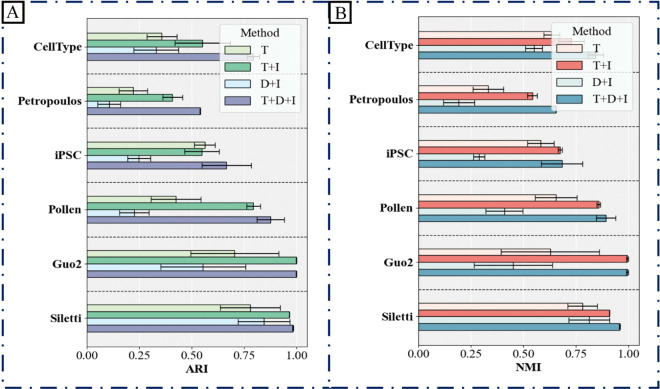
Ablation study evaluating the contribution of D3Impute modules. Bar plots display the clustering performance, measured by (A) Adjusted Rand Index (ARI) and (B) Normalized Mutual Information (NMI), of four different pipeline configurations across six scRNA-seq datasets. The evaluated configurations include: transformation only (T), transformation with imputation (T+I), discrimination with imputation (D+I), and the complete framework (T+D+I). The superior performance of the complete T+D+I pipeline underscores the necessity and synergistic interaction of all three modules.

Clustering performance was evaluated on six real scRNA-seq datasets, with results expressed as the mean ± standard deviation of the Adjusted Rand Index (ARI) and Normalized Mutual Information (NMI) over ten independent runs (Fig 7A: ARI, Fig 7B: NMI). The complete T+D+I configuration consistently achieved the best performance across all datasets, demonstrating a strong synergistic effect among the three modules. In contrast, the D+I combination performed the worst, indicating that the discrimination module is highly sensitive to technical noise in the raw data and prone to misclassification in the absence of appropriate transformation. This underscores the critical role of the transformation module in mitigating technical artifacts and enhancing discrimination accuracy.

Collectively, these ablation results validate the functional design and interdependence of the individual modules in D3Impute and substantiate the rationale for employing the integrated three-module pipeline.

### Downstream analysis

In this section, we performed a comprehensive evaluation of our method against 12 state-of-the-art approaches through three fundamental single-cell RNA sequencing (scRNA-seq) analytical tasks: (1) cell clustering, (2) pseudotemporal trajectory inference, and (3) differential gene expression analysis.

#### Comparative evaluation of cell clustering performance.

We conducted systematic benchmarking of D3Impute against 12 leading scRNA-seq imputation methods using six well-annotated datasets with established biological ground truth: Siletti (human brain), Guo2 (human primordial germ cells), Pollen (human cerebral cortex), iPSC (human induced pluripotent stem cells), Petropoulos (human preimplantation embryonic), and CellType (human embryonic stem cell-derived progenitors). Cluster quality was quantitatively assessed using two established metrics, ARI and NMI.

**Fig 8:**
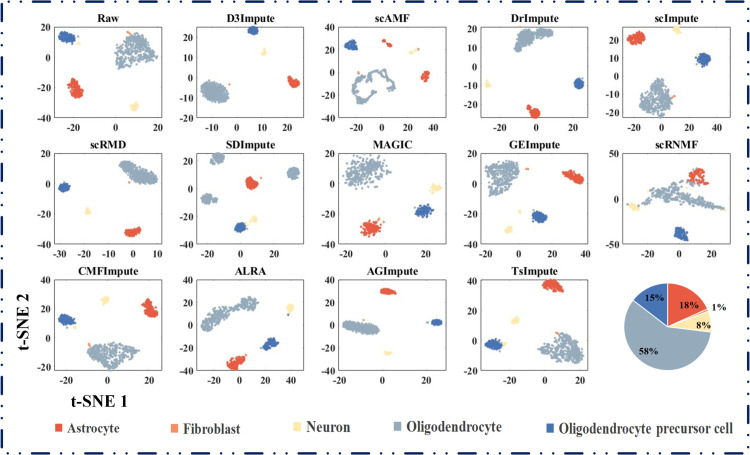
Cell clustering in Siletti dataset before and after imputation. Two-dimensional visualization using multiple algorithms highlights structural changes in cell populations. t-SNE projections were generated from raw and imputed expression matrices. Each point represents a single cell, colored by its annotated cell type. The pie chart in the lower right corner summarizes the cell-type composition of the Siletti dataset, which comprises five distinct neural subtypes.

As demonstrated in Table 9 and Table 10, D3Impute achieved superior clustering performance on four datasets: Siletti (ARI: 0.9822±0.0024, NMI: 0.9590±0.0030), Guo2 (ARI: 0.9992±0.0024, NMI: 0.9963±0.0050), Pollen (ARI: 0.8761±0.0656, NMI: 0.8933±0.0458), and Petropoulos (ARI: 0.5399±0.0001, NMI: 0.6554±0.0001). Notably, ALRA showed optimal performance on iPSC (ARI: 0.8420±0.0225, NMI: 0.8317±0.0169) and CellType (ARI: 8672±0.1004, NMI: 0.8916±0.0410). The aggregated analysis reveals that D3Impute maintains significantly better mean performance with lower variance compared to all alternatives, including its closest competitor, ALRA. t-SNE projections (Fig 8) confirmed that D3Impute preserves original biological distributions while generating more distinct cell-type clusters in the Siletti dataset. This pattern was consistently observed across all evaluated datasets (Supporting information S10 Fig–S14 Fig).

**Table 9:** Clustering performance (ARI mean ± standard deviation) over ten runs for imputation methods across six datasets.

Method	Siletti	Guo2	Pollen	iPSC	Petropoulos	CellType
Raw	0.7869±0.1805	0.8096±0.4258	0.2257±0.1501	0.2406±0.2096	0.3626±0.0453	0.4766±0.0755
D3Impute	**0.9822±0.0024**	**0.9992±0.0011**	**0.8761±0.0656**	0.6657±0.1167	**0.5399±0.0001**	0.7931±0.0291
scAMF	0.9615±0.0000	0.7421±0.0000	0.4358±0.1262	0.6082±0.1029	0.4363±0.0194	0.7526±0.0000
DrImpute	0.8485±0.1369	0.8096±0.4258	0.4109±0.1267	0.6169±0.1289	0.3721±0.0263	0.5962±0.1789
scImpute	0.8805±0.1595	0.2837±0.2277	0.1605±0.0370	0.1767±0.1343	0.0649±0.0266	0.2252±0.0941
scRMD	0.7530±0.1970	0.8048±0.4366	0.4885±0.1257	0.3464±0.2448	0.3587±0.0825	0.6299±0.1781
SDImpute	0.5691±0.0908	0.7398±0.4285	0.6043±0.1472	0.5703±0.0682	0.3646±0.0332	0.5304±0.1691
MAGIC	0.2978±0.1973	0.1644±0.2464	0.0904±0.0244	0.0321±0.0270	0.0619±0.0236	0.1531±0.1006
GEImpute	0.6840±0.1543	0.1575±0.2514	0.2093±0.0762	0.2588±0.1603	0.0911±0.0255	0.2258±0.0986
scRNMF	0.5434±0.0400	0.2562±0.1426	0.1132±0.0172	0.2620±0.1147	0.0556±0.0353	0.3111±0.0479
CMFImpute	0.7659±0.1980	0.3188±0.2558	0.1932±0.0857	0.2761±0.1422	0.0747±0.0564	0.2264±0.1167
ALRA	0.6763±0.1482	0.9499±0.0001	0.8740±0.1189	**0.8420±0.0225**	0.3895±0.0161	**0.8672±0.1004**
AGImpute	0.6769±0.2625	0.9833±0.0001	0.8346±0.0236	0.3851±0.1082	0.4062±0.0107	0.7194±0.0767
TsImpute	0.9002±0.0712	0.7421±0.0219	**-**	0.1551±0.0840	0.1244±0.0259	**-**

**Table 10:** Clustering performance (NMI mean ± standard deviation) over ten runs for imputation methods across six datasets.

Method	Siletti	Guo2	Pollen	iPSC	Petropoulos	CellType
Raw	0.7796±0.1595	0.8068±0.4319	0.4171±0.1951	0.2735±0.1765	0.4722±0.0355	0.6878±0.0416
D3Impute	**0.9590±0.0030**	**0.9963±0.0050**	0.8933±0.0458	0.6839±0.0983	**0.6554±0.0001**	0.8446±0.0362
scAMF	0.9240±0.0001	0.6280±0.0001	0.6712±0.1093	0.6795±0.0589	0.5411±0.0238	0.8423±0.0000
DrImpute	0.7406±0.2353	0.8068±0.4319	0.6729±0.0981	0.6554±0.0876	0.4920±0.0365	0.7490±0.0923
scImpute	0.8537±0.1163	0.1997±0.1665	0.3719±0.0569	0.2135±0.1078	0.2089±0.1703	0.4970±0.0637
scRMD	0.7511±0.2163	0.8034±0.4396	0.7302±0.0774	0.4297±0.2103	0.3849±0.1305	0.7786±0.0722
SDImpute	0.6751±0.0559	0.7173±0.4302	0.7759±0.0856	0.5918±0.0792	0.4827±0.0337	0.6756±0.1089
MAGIC	0.3241±0.1833	0.1286±0.1993	0.2281±0.0377	0.0554±0.0396	0.1665±0.1399	0.2768±0.1324
GEImpute	0.6784±0.1179	0.1229±0.2057	0.4111±0.0734	0.2824±0.1320	0.1703±0.0521	0.4594±0.1671
scRNMF	0.5652±0.0533	0.1926±0.1080	0.2549±0.0204	0.3093±0.0843	0.0844±0.0314	0.5072±0.0549
CMFImpute	0.7467±0.1821	0.2481±0.2046	0.3694±0.0758	0.2971±0.1211	0.1383±0.0369	0.5317±0.0121
ALRA	0.7645±0.0988	0.8970±0.0000	**0.9207±0.0527**	**0.8317±0.0169**	0.4962±0.0104	**0.8916±0.0410**
AGImpute	0.7243±0.1789	0.9585±0.0002	0.8875±0.0409	0.4985±0.0964	0.5036±0.0065	0.7908±0.0454
TsImpute	0.8448±0.0500	0.6284±0.0239	**-**	0.2194±0.0659	0.2237±0.0225	**-**

#### Comparative evaluation of pseudotemporal trajectory reconstruction.

Beyond characterizing cellular heterogeneity through clustering, trajectory inference enables the investigation of dynamic biological processes, including development, differentiation, and cellular responses. This approach reconstructs continuous paths through transcriptional space by minimizing gene expression variations between adjacent cells, representing cellular transitions through pseudotemporal ordering. Following established benchmarking protocols from scMOO [44], we evaluated D3Impute and other 12 advanced imputation algorithms using the iPSC dataset with experimentally validated temporal labels as ground truth. Our assessment framework incorporates topological accuracy and POS and Kendall’s rank correlations (Kendall).

**Fig 9:**
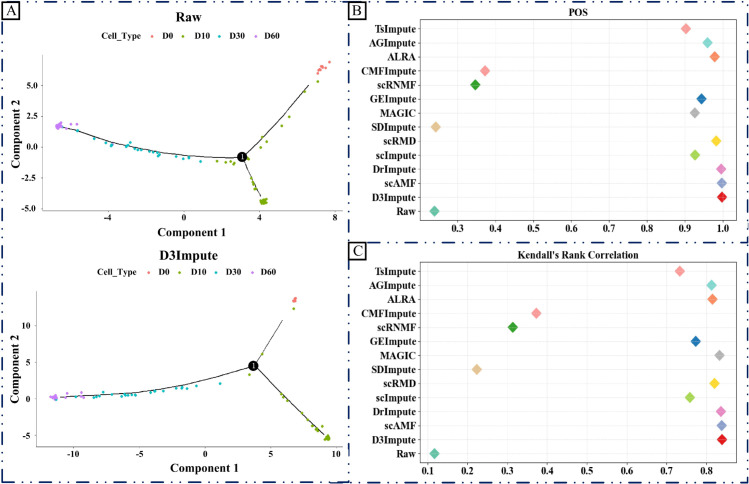
Evaluation of imputation methods through pseudotime analysis by Monocle 2 on the iPSC dataset. (A) Visualization of lineages reconstructed from the observed and imputed data. Cells are embedded into a 2D space using reversed graph embedding. Different colors correspond to different cell stages. (B) The POS of different methods. (C) Kendall’s rank correlation scores of different methods. Different colors correspond to different imputation methods.

As demonstrated in Fig 9B and 9C, D3Impute achieved superior performance (POS:0.9985,Kendall:0.8392) compared to scAMF (POS:0.9980,Kendall:0.8152) and DrImpute (POS:0.9963,Kendall:0.8364). Notably, while all three methods show high concordance with the experimental timeline (>99.6% position accuracy), D3Impute maintains the strongest correlation with the gold-standard temporal ordering. These results position D3Impute as particularly suitable for developmental studies requiring precise trajectory inference.

#### Comparative performance in differential gene expression analysis.

The identification of differentially expressed genes (DEGs) represents a fundamental analytical task in scRNA-seq studies, enabling the characterization of molecular mechanisms driving cellular heterogeneity and state transitions. Our evaluation framework employs bulk RNA-seq data as the gold standard reference, given its inherent robustness against dropout artifacts [40].

**Fig 10:**
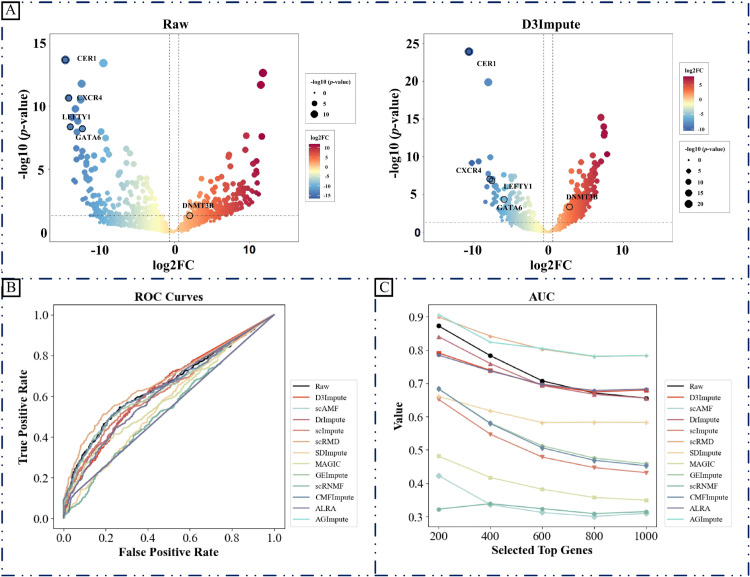
Evaluation of imputation methods through differential expression analysis on CellType dataset (H1-DEC). (A) Volcano plots comparing DEGs identified from raw and D3Impute-imputed data. Black circles indicate biologically validated enriched genes (CER1, CXCR4, LEFTY1, GATA6, DNMT3B), used to assess consistency of imputation-enhanced differential expression. (B) ROC curves illustrating the ability of different imputation methods to recover DEGs, using bulk RNA-seq as reference. Curves are generated by ranking genes from imputed data and comparing them against the top 1000 bulk-derived DEGs (based on adjusted *p*-values). (C) AUC scores comparing the DEG detection performance of different imputation methods. Scores are calculated using bulk-derived reference sets of the top 200, 400, 600, 800, and 1000 DEGs, enabling evaluation across varying detection thresholds.

When the top 200, 400, and 600 genes are chosen, Fig 10C demonstrates that D3Impute performs worse than the Raw dataset regarding the AUC scores. As soon as the top 800 and top 1000 are chosen, D3Impute begins to overcome the raw dataset. Notably, the performance of AGImpute, scRMD, ALRA, and D3Impute consistently ranks in the top three in most situations. Both AGImpute, ALRA, and D3Impute use zero inference. This indicates that the identification of zero values via filling is an effective strategy for enhancing performance, particularly in DE analysis.

Fig 10A is a volcano graphic that displays the raw and imputed data. Compared to the Raw dataset, the imputed data utilizing D3Impute can more accurately identify highly expressed genes. Research conducted by Chen and Zhou [40] indicates that DEG cells are abundant in genes including CER1 (raw data’s –log(*p*-value): 13.6676, imputed data’s –log(*p*-value): 21.1864), CXCR4 (raw data’s –log(*p*-value): 10.6459, imputed data’s –log(*p*-value): 10.3288), LEFTY1 (raw data’s –log(*p*-value): 8.3635, imputed data’s –log(*p*-value): 10.0325), GATA6 (raw data’s –log(*p*-value): 8.2027, imputed data’s –log(*p*-value): 7.6635), and DNMT3A (raw data’s –log(*p*-value): 1.3018, imputed data’s –log(*p*-value): 2.50724). Our technique exhibits superior –log(*p*-value) values for CER1 and DNMT3A in comparison to the raw data.

### Computational resource analysis

To systematically evaluate the computational efficiency and resource consumption of various imputation algorithms across different data scales and biological contexts, we conducted a complementary two-pronged experimental analysis. This analysis encompasses cross-dataset benchmarking and a scalability assessment under controlled conditions.

**Fig 11:**
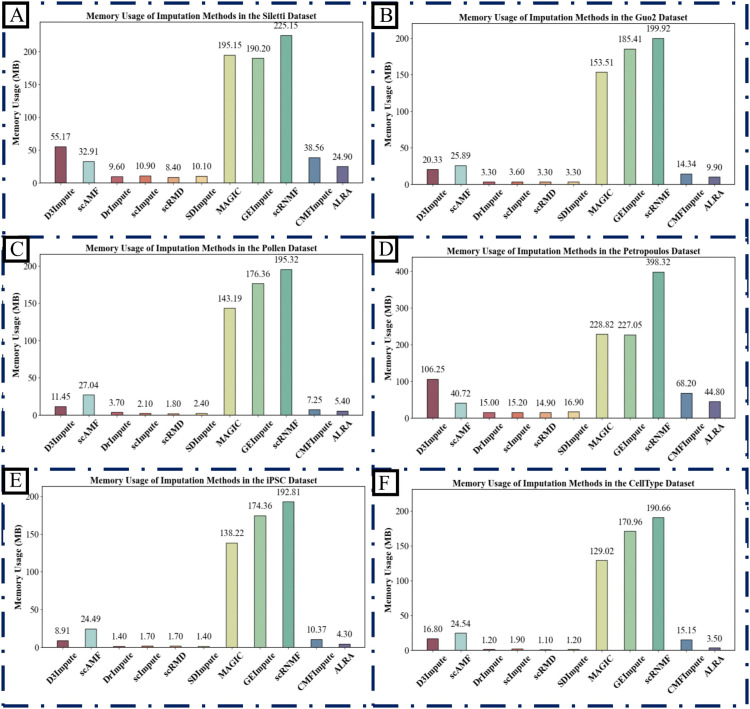
Computational efficiency benchmarking of imputation algorithms. Main thread runtime of 12 imputation methods was evaluated across six scRNA-seq datasets with diverse biological contexts: (A) Siletti, (B) Guo2, (C) Pollen, (D) iPSC, (E) Petropoulos, and (F) CellType. Each subplot illustrates the computational time required by each method on the corresponding dataset, providing a systematic assessment of their efficiency and scalability.

We first compared the runtime performance of 13 leading imputation algorithms on six representative scRNA-seq datasets (Siletti, Guo2, Pollen, iPSC, Petropoulos, and CellType) from diverse tissues. This experiment aimed to benchmark their general efficiency and sensitivity to data heterogeneity and sparsity. As detailed in Fig 11 and Supporting information S5 Table, significant disparities in runtime were observed. AGImpute consistently required over 300 seconds across all six datasets, a duration substantially longer than other methods, and thus its results are omitted from the figures for clarity. Overall, D3Impute, scRMD, MAGIC, GEImpute, and ALRA demonstrated stable and efficient performance across all datasets, indicating robust generalizability in diverse biological environments. Peak memory usage for each algorithm on the six datasets is profiled in Supporting information S15 Fig and S6 Table. The results reveal distinct memory consumption patterns. MAGIC, GEImpute, and scRNMF exhibited significantly higher memory footprints compared to other algorithms. In contrast, D3Impute, scRMD, ALRA, and most other methods maintained low memory usage across all datasets, showcasing superior resource efficiency for standard analytical workloads.

To quantitatively assess the scalability of each algorithm with increasing data size, we adopted a strategy inspired by the scalability evaluation in scRNMF. We generated six subsets of varying scales from the large-scale dataset GSM4505405 (containing 110828 cells and 22966 genes). After preprocessing and selecting 2000 highly variable genes, we randomly sampled 2000, 4000, 6000, 8000, 10000, and 20000 cells to create the subsets. The runtime and peak memory usage for all 13 algorithms on these subsets are documented in Supporting information S16 Fig, S7 Table, and S8 Table. AGImpute exceeded 6 hours of runtime on all subset sizes and is excluded from the figures. The results indicate that the runtime of D3Impute, DrImpute, scImpute, SDImpute, and TsImpute increased exponentially with data size, suggesting potential efficiency bottlenecks when processing ultra-large-scale data. In contrast, the runtime of CMFImpute and scAMF scaled linearly, demonstrating more favorable scalability. Furthermore, the memory usage of all algorithms increased linearly with data size, aligning with expected resource consumption patterns for in-memory computations.

## Discussion

D3Impute establishes a robust computational framework for addressing the pervasive challenge of dropout events in single-cell RNA sequencing (scRNA-seq) data. Through comprehensive evaluation across six biologically diverse datasets, we demonstrate its consistent superiority over existing methods in three key analytical tasks: differential gene expression analysis, cell type clustering, and pseudotemporal trajectory reconstruction. Quantitative assessments confirm that D3Impute enhances clustering accuracy, improves trajectory inference reliability, and increases sensitivity in detecting differentially expressed genes.

(1) Necessity of data-specific transformations.

**Fig 12:**
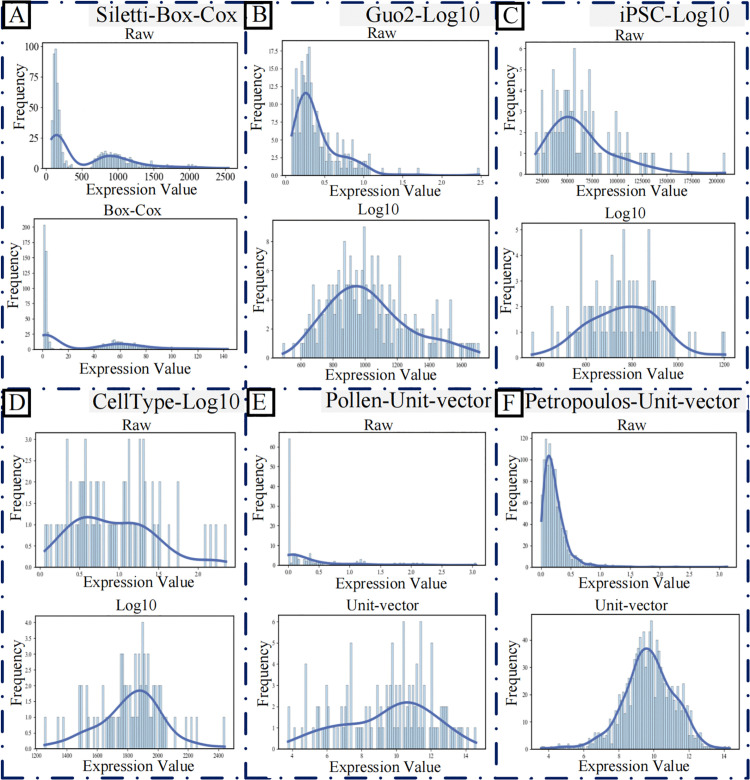
Comparative visualization of per-cell expression distributions before and after optimal transformation across six datasets. Histograms show the frequency distribution of total transcript counts per cell, binned along the x-axis. The y-axis indicates the number of cells falling within each expression bin. Each panel illustrates the effect of a dataset-specific transformation in reducing skewness and improving distribution symmetry. (A) Box-Cox transformation for the Siletti dataset. (B) Log10 transformation for the Guo2 dataset. (C) Log10 transformation for the iPSC dataset. (D) Log10 transformation for the CellType dataset. (E) Unit-vector transformation for the Pollen dataset. (F) Unit-vector transformation for the Petropoulos dataset.

The substantial variation in initial skewness coefficients (SK=0.6–4.4) across datasets underscores the necessity for data-specific preprocessing strategies. Our transformation framework successfully normalized all distributions to SK≤0.4, approximating Gaussian characteristics and significantly improving cluster separability (Table 8 and Fig 12). Distinct transformation rules emerged based on distribution patterns: Box-Cox transformation optimally handled bimodal distributions (e.g., Siletti, initial SK=1.1); Log10 transformation effectively normalized moderately skewed unimodal distributions (|SK|<2.5); and Unit-vector transformation was essential for severely skewed distributions (|SK|≥2.5) to reduce skewness while preserving biological relevance. These findings provide a quantitative, data-driven protocol for preprocessing strategy selection, demonstrating broad applicability across diverse single-cell genomics contexts.

To further evaluate imputation effects on expression distributions, we performed comparative visualization of gene expression histograms before and after imputation (S17 Fig–S2 Fig). These analyses revealed that D3Impute successfully recovers dropout events while maintaining original distribution shapes without introducing artificial distortion. The preserved distributional integrity underscores D3Impute’s capacity to balance accurate imputation with biological fidelity.

(2) Biological significance of the joint low-dimensional embedding space

**Fig 13:**
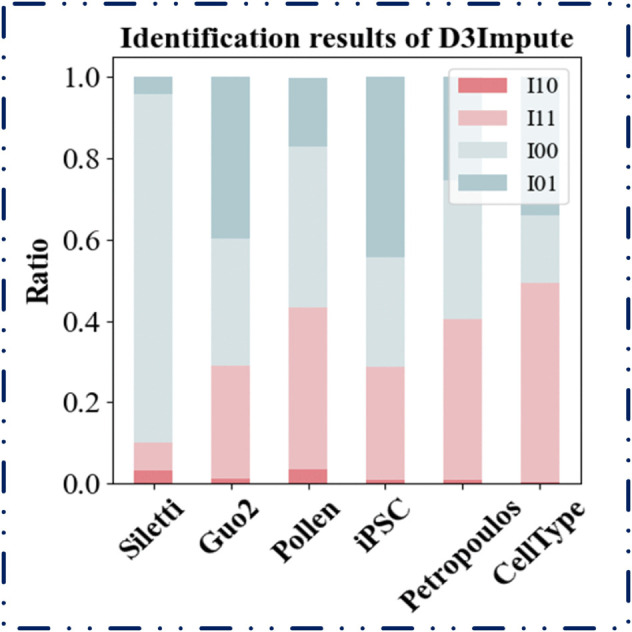
Stacked bar chart showing the proportional distribution of zero-type predictions across six benchmark datasets. Each bar represents the composition of four prediction categories derived from an element-wise comparison between the observed indicator matrix *I*_0_ and the reconstructed matrix I^*^, across all gene-cell positions. The stacked segments correspond to: (i) *I*_10_ (False negatives): observed expression misclassified as zero (*I*_0*ij*_ = 1 and $I_{ij}^{*}=0$); (ii) *I*_11_ (True positives): observed expression correctly retained (*I*_0*ij*_ = 1 and $I_{ij}^{*}\neq 0$ ); (iii) *I*_01_ (Non-biological zeros): unobserved expression predicted as present (*I*_0*ij*_ = 0 and $I_{ij}^{*}\neq 0$), requiring imputation; (iv) *I*_00_ (Biological zeros): unobserved expression correctly predicted as absent (*I*_0*ij*_ = 0 and $I_{ij}^{*}=0$).

The biological relevance of our joint low-dimensional embedding is demonstrated by its capacity to capture coherent gene–cell co-expression patterns. By comparing the original indicator matrix *I*_0_ and the reconstructed matrix I^*^, we identified positions where *I*_0*ij*_ = 0 and $I_{ij}^{*}\neq 0$ as high-confidence, biologically expressed genes that were obscured by dropout events. Comprehensive evaluation across six datasets (Fig 13) revealed extremely low frequencies (<0.0343) of potential false negatives (*I*_0*ij*_ = 1 and $I_{ij}^{*}=0$), underscoring the robustness of our approach.

**Fig 14:**
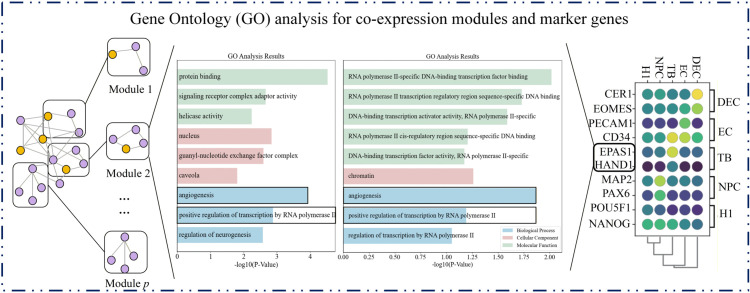
Biological validation of latent space representations in the CellType dataset. This figure illustrates the biological relevance of the unified low-dimensional embedding space constructed by jointly mapping gene–gene and cell–cell networks. The left panel shows gene co-expression modules (Module 1, Module 2, ⋯, Module *p*) identified from the embedded gene network. The right panel displays marker genes for five differentiation-related cell types (DEC, EC, TB, NPC, H1). The center panel presents results from gene ontology (GO) enrichment analysis, performed separately on gene modules and cell-type marker genes, enabling functional comparison between network-derived modules and known cell-type signatures.

To further interpret the latent space, we analyzed differentiation-related cell types (DEC, EC, NPC, H1, TB) from the CellType dataset. Using WGCNA, we identified co-expression modules and performed GO enrichment analysis. Notably, Module 2 (158 genes) exhibited strong functional concordance with TB cell marker genes (Fig 14), enriching for angiogenesis (GO:0001525, *p*-value: $1.15\;\times \;10^{-4}$) and positive regulation of RNA polymerase II (GO:0045944, *p*-value: $1.32\;\times \;10^{-3}$)—processes consistent with known TB markers EPAS1 and HAND1. These results confirm that the embedding space captures biologically meaningful regulatory architecture, aligning gene modules with established cell-type signatures.

(3) Enhanced robustness through shared nearest neighbor analysis

**Fig 15:**
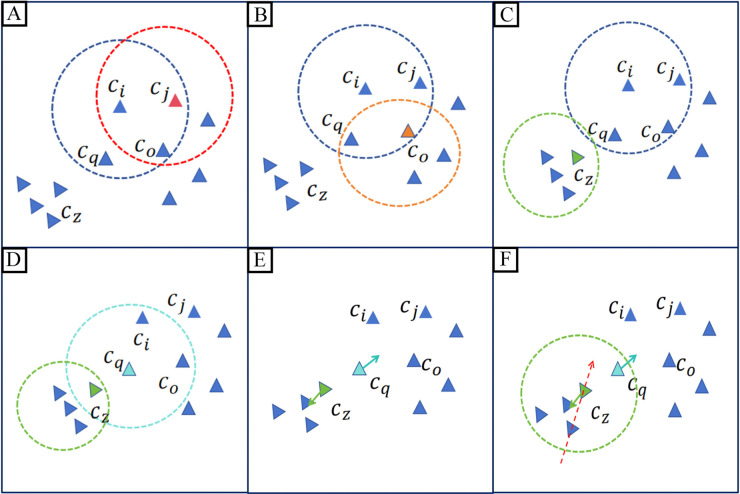
Efficacy of density-guided imputation engine. (A) Scenario I: Red/blue dashed circles denote neighborhoods of $c_{j}/c_{i}$, respectively, with overlap indicating their SNNs. (B) Scenario II: Orange circles highlight the transitional neighborhood *c*_*o*_, and the SNN overlaps with *c*_*i*_. (C) Scenario III: Green circles demarcate discrete cell state *c*_*z*_ neighborhoods. (D) Comparative spatial configuration of boundary cell *c*_*q*_ (aqua circle). (E) Centroid-directed migration vectors for *c*_*z*_ and *c*_*q*_. (F) Misclassification-induced deviation (red arrow) when *c*_*q*_ is erroneously assigned to *c*_*z*_’s neighborhood.

We further validated the geometric fidelity of our approach by analyzing three canonical topological scenarios using shared nearest neighbor (SNN) relationships. Scenario I captures homogeneous cell neighborhoods with high SNN similarity, Scenario II identifies transitional states within continuous biological processes, and Scenario III maintains strict segregation between discrete cell types. While Euclidean distance performed adequately in conventional clustering tasks (Fig 15A–C), critical divergences emerged at cluster boundaries (Fig 15D). In heterogeneous populations with density variations, Euclidean metrics misassigned boundary cell *c*_*q*_ based on absolute proximity, whereas SNN preserved correct cell affiliations through relative neighborhood overlap. This highlights SNN’s superior robustness in handling local density fluctuations and boundary ambiguity—an essential attribute for analyzing developmental trajectories or rare cell subpopulations.

(4) Downstream analytical gains: task-specific utility of imputation

The impact of imputation on scRNA-seq data analysis is highly dependent on the specific analytical task. Using a systematic benchmarking framework, we evaluated 12 imputation methods across three core tasks—cell clustering, pseudotime inference, and differential expression analysis—on six representative datasets.

**Fig 16:**
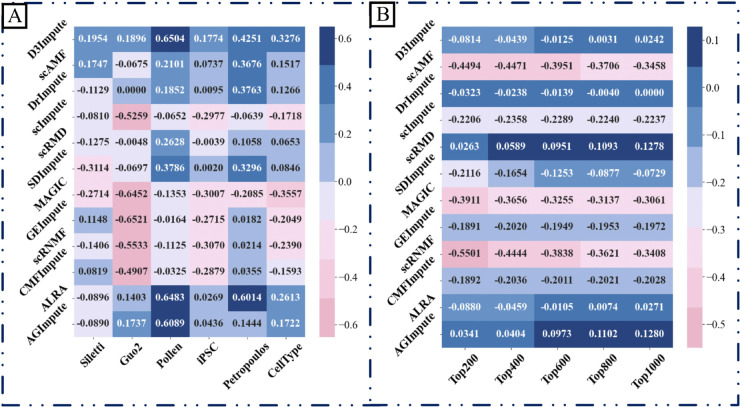
Impact of imputation on the performance of downstream analytical tasks. (A) Heatmap showing the performance changes in cell clustering before and after imputation. (B) Heatmap showing the AUC gains on the CellType dataset (H1-DEC) before and after imputation.

For cell clustering, performance gains were closely tied to the intrinsic structural clarity of the data, as quantified by the silhouette coefficient (SC) of the raw data. In datasets with well-defined separation (e.g., Guo2, SC = 0.8879; Siletti, SC =0.2346), imputation provided minimal improvement in the Adjusted Rand Index (ARI). In contrast, for datasets with overlapping or poorly separated clusters (e.g., Pollen, iPSC, Petropoulos, and CellType), imputation consistently enhanced cluster discrimination by recovering expression signals essential for distinguishing cell types (Fig 16A).

In pseudotime inference, a global structure task, imputation proved particularly beneficial. On the structurally complex iPSC dataset, most methods significantly improved the Pseudo-temporal Ordering Score (POS), underscoring the role of imputation in reconstructing biological continua from data with ambiguous cellular relationships (S23 Fig).

In differential expression analysis, a nuanced profile emerged. When evaluated against a bulk RNA-seq gold standard (CellType dataset), imputation yielded only marginal gains for the most pronounced differentially expressed genes (Top 200–400; Fig 16B). However, for broader gene sets (Top 600–1000), it significantly improved AUC, highlighting its role in enhancing statistical power to detect genes with modest fold-changes that are otherwise obscured by data sparsity.

In summary, these findings support a pragmatic framework for imputation use: strongly recommended for global structure recovery, context-dependent for clustering, and most valuable for improving sensitivity in differential expression beyond the most extreme markers.

(5) Practical guidelines for bulk RNA-seq reference selection

We recognize that obtaining perfectly matched bulk RNA-seq data—sharing the same tissue, developmental stage, and experimental conditions—is often challenging. Moreover, the absence of a universal “gold standard” for assessing bulk data quality complicates the prediction of how reference variations influence D3Impute’s performance.

**Fig 17:**
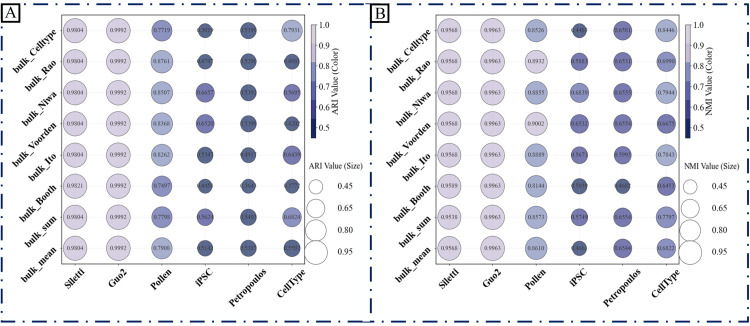
Clustering performance of D3Impute across 48 scRNA-seq–bulk reference combinations for practical reference selection. (A) Impact of bulk RNA-seq selection on D3Impute’s post-imputation clustering accuracy (ARI). (B) Impact of bulk RNA-seq selection on D3Impute’s post-imputation clustering accuracy (NMI).

To provide actionable guidance, we systematically evaluated the method across 48 scRNA-seq–bulk combinations, comprising six scRNA-seq datasets and eight bulk references (six real, two pseudo-bulk). Using ARI and NMI as metrics (Fig 17), we derived the following operational guidelines:

- Optimal performance: Using bulk RNA-seq from the same species and tissue source (e.g., Siletti-Booth) yields the highest ARI and NMI, significantly outperforming other combinations.
- Reliable alternative: When matched data are unavailable, bulk data from pluripotent stem cells (e.g., Niwa) provide stable, high-quality imputation.
- Practical fallback: In the absence of external bulk data, constructing pseudo-bulk references (e.g., bulk-mean or bulk-sum) from the scRNA-seq data itself still substantially improves clustering performance.

A key contribution of D3Impute is its dual-network embedding strategy, which synergistically leverages bulk and single-cell RNA-seq data to distinguish biological from non-biological zeros. Unlike methods that rely solely on scRNA-seq data (e.g., scImpute, scRMD, ALRA), our cross-technique framework preserves inter-gene relationships observed in bulk data, enabling more accurate identification of dropout events. This biologically informed design addresses the critical challenge of maintaining genuine expression relationships obscured by technical zeros.

Furthermore, the proposed dropout-aware discriminator is modular and flexible, allowing for integration with various imputation algorithms to enhance their performance. These features ensure that D3Impute remains widely applicable across diverse research scenarios, even when ideal reference data are unavailable.

## Conclusion

D3Impute establishes a biologically grounded framework for addressing the critical challenge of dropout events in scRNA-seq data analysis. Through systematic evaluation across six biologically diverse datasets, we demonstrate that our method consistently outperforms existing approaches in three fundamental analytical tasks: differential expression analysis, cell type clustering, and pseudotemporal trajectory reconstruction. The key innovation lies in our dual-network embedding strategy that synergistically leverages bulk RNA-seq and scRNA-seq data to accurately distinguish biological zeros from technical artifacts—a capability that directly translates to enhanced performance in downstream analyses.

Our investigation reveals several important practical insights. First, the effectiveness of imputation is highly dependent on the specific analytical task and dataset characteristics. While D3Impute significantly improves clustering resolution for datasets with poorly defined structures, it proves particularly valuable for reconstructing biological continua in pseudotemporal ordering. Second, our systematic evaluation of reference selection strategies provides clear guidelines for real-world applications, demonstrating that robust performance can be achieved even without perfectly matched bulk data through the use of pluripotent stem cell references or pseudo-bulk alternatives.

Beyond technical performance, the biological relevance of our approach is evidenced by its ability to maintain functional coherence in gene co-expression modules while accurately recovering dropout events. The shared nearest neighbor analysis further confirms the method’s robustness in handling complex cellular neighborhoods and boundary cases. As single-cell technologies continue to evolve, we believe that biologically informed frameworks like D3Impute will play an increasingly crucial role in unlocking the full potential of scRNA-seq data for deciphering cellular heterogeneity across diverse research and clinical applications.

## Supporting information

S1 Text The detail of update rules and convergence for dropout-aware discrimination. (PDF)

S1 FigParameter optimization analysis for D3Impute on the Guo2 dataset.(A) The waterfall chart illustrates sixteen parameter combinations of *β* and *λ* (where $\lambda =\lambda_{c}=\lambda_{g}$), with optimal model performance achieved at β=0.0001 and λ=0.0001. (B) Subsequent parameter tuning identified *k* = 16. (C) Subsequent parameter tuning identified *p* = 10.(TIFF)

S2 FigParameter optimization analysis for D3Impute on the Pollen dataset.(A) The waterfall chart illustrates sixteen parameter combinations of *β* and *λ* (where $\lambda =\lambda_{c}=\lambda_{g}$), with optimal model performance achieved at β=0.1 and λ=0.1. (B) Subsequent parameter tuning identified *k* = 7. (C) Subsequent parameter tuning identified *p* = 10.(TIFF)

S3 FigParameter optimization analysis for D3Impute on the iPSC dataset.(A) The waterfall chart illustrates sixteen parameter combinations of *β* and *λ* (where $\lambda =\lambda_{c}=\lambda_{g}$), with optimal model performance achieved at β=0.1 and λ=0.1. (B) Subsequent parameter tuning identified *k* = 7. (C) Subsequent parameter tuning identified *p* = 10.(TIFF)

S4 FigParameter optimization analysis for D3Impute on the Petropoulos dataset.(A) The waterfall chart illustrates sixteen parameter combinations of *β* and *λ* (where $\lambda =\lambda_{c}=\lambda_{g}$), with optimal model performance achieved at β=0.01 and λ=0.01. (B) Subsequent parameter tuning identified *k* = 10. (C) Subsequent parameter tuning identified *p* = 10.(TIFF)

S5 FigParameter optimization analysis for D3Impute on the CellType dataset.(A) The waterfall chart illustrates sixteen parameter combinations of *β* and *λ* (where $\lambda =\lambda_{c}=\lambda_{g}$), with optimal model performance achieved at β=0.01 and λ=0.01. (B) Subsequent parameter tuning identified *k* = 6. (C) Subsequent parameter tuning identified *p* = 10.(TIFF)

S6 Fig Benchmarking results under a 70% dropout rate. (A) 2D visualization of the masked dataset (seed = 88) before and after imputation, illustrating the restoration of data structure. (B) Pearson correlation coefficient (PCC) between the imputed and ground truth expression. (C) Root mean square error (RMSE) of imputed values. (D) Proportion of correctly recovered non-biological zeros. (E) Proportion of falsely imputed biological zeros.
(TIFF)

S7 Fig Benchmarking results under a 80% dropout rate. (A) 2D visualization of the masked dataset (seed = 55) before and after imputation. (B) Pearson correlation coefficient (PCC) between the imputed and ground truth expression. (C) Root mean square error (RMSE) of imputed values. (D) Proportion of correctly recovered non-biological zeros. (E) Proportion of falsely imputed biological zeros.
(TIFF)

S8 Fig ARI-based clustering performance across six scRNA-seq datasets. (A) Siletti, (B) Guo2, (C) Pollen, (D) iPSC, (E) Petropoulos, and (F) CellType.
(TIFF)

S9 Fig NMI-based clustering performance across six scRNA-seq datasets. (A) Siletti, (B) Guo2, (C) Pollen, (D) iPSC, (E) Petropoulos, and (F) CellType.
(TIFF)

S10 Fig Two-dimensional visualization of cell clustering in the Guo2 dataset before and after imputation using multiple algorithms. t-SNE projections were generated from raw and imputed expression matrices. Each point represents a single cell, colored by its annotated cell type. The pie chart in the lower right corner summarizes the cell-type composition of the Guo2 dataset, which comprises two distinct subtypes.
(TIFF)

S11 Fig Two-dimensional visualization of cell clustering in the Pollen dataset before and after imputation using multiple algorithms. t-SNE projections were generated from raw and imputed expression matrices. Each point represents a single cell, colored by its annotated cell type. The pie chart in the lower right corner summarizes the cell-type composition of the Pollen dataset, which comprises eight distinct subtypes.
(TIFF)

S12 Fig Two-dimensional visualization of cell clustering in the iPSC dataset before and after imputation using multiple algorithms. t-SNE projections were generated from raw and imputed expression matrices. Each point represents a single cell, colored by its annotated cell type. The pie chart in the lower right corner summarizes the cell-type composition of the iPSC dataset, which comprises four distinct subtypes.
(TIFF)

S13 Fig Two-dimensional visualization of cell clustering in the Petropoulos dataset before and after imputation using multiple algorithms. t-SNE projections were generated from raw and imputed expression matrices. Each point represents a single cell, colored by its annotated cell type. The pie chart in the lower right corner summarizes the cell-type composition of the Petropoulos dataset, which comprises five distinct subtypes.
(TIFF)

S14 Fig Two-dimensional visualization of cell clustering in the CellType dataset before and after imputation using multiple algorithms. t-SNE projections were generated from raw and imputed expression matrices. Each point represents a single cell, colored by its annotated cell type. The pie chart in the lower right corner summarizes the cell-type composition of the CellType dataset, which comprises seven distinct neural subtypes.
(TIFF)

S15 Fig Computational efficiency benchmarking of imputation algorithms. Peak memory usage comparison of imputation algorithms across six scRNA-seq datasets with diverse biological contexts: (A) Siletti dataset; (B) Guo2 dataset; (C) Pollen dataset; (D) Petropoulos dataset; (E) iPSC dataset; (F) CellType dataset. Each subplot shows the peak memory usage of 12 imputation algorithms on the corresponding dataset, evaluating the resource consumption characteristics of the algorithms under different biological backgrounds.
(TIFF)

S16 Fig Scalability assessment: Runtime performance analysis of imputation algorithms under different data scales. (A) Runtime comparison plot, showing the main thread runtime of each algorithm for cell sizes of 2000, 4000, 6000, 8000, 10000, and 20000, displaying data within 1000 seconds only; (B) Memory usage comparison plot, showing the peak memory usage of each algorithm under different data scales, displaying data below 2000 MB only.
(TIFF)

S17 Fig Gene expression distribution before and after imputation on the Siletti dataset. (TIFF)

S18 Fig Gene expression distribution before and after imputation on the Guo2 dataset. (TIFF)

S19 Fig Gene expression distribution before and after imputation on the Pollen dataset. (TIFF)

S20 Fig Gene expression distribution before and after imputation on the iPSC dataset. (TIFF)

S21 Fig Gene expression distribution before and after imputation on the Petropoulos dataset. (TIFF)

S22 Fig Gene expression distribution before and after imputation on the CellType dataset. (TIFF)

S23 Fig Bar plot showing the performance changes in pseudotemporal Ordering Score (POS) on the iPSC dataset before and after imputation. (TIFF)

S1 Table ARI comparison of direct imputation methods with and without dropout-aware discriminator across six datasets. (XLSX)

S2 Table NMI comparison of direct imputation methods with and without dropout-aware discriminator across six datasets. (XLSX)

S3 Table ARI comparison of the filling effects of two imputation paradigms across six datasets. (XLSX)

S4 Table NMI comparison of the filling effects of two imputation paradigms across six datasets. (XLSX)

S5 Table Runtime (s) of 12 algorithms across six datasets. (XLSX)

S6 Table Peak memory usage (MB) of 12 algorithms across six datasets. (XLSX)

S7 Table Runtime (s) of 12 algorithms across datasets of varying sizes. (XLSX)

S8 Table Peak memory usage (MB) of 12 algorithms across datasets of varying sizes. (XLSX)

S9 Table Skewness coefficients of total expression distributions across six datasets before imputation, after data transformation, and after imputation. (XLSX)
