# Supplementary material for: D3Impute: Dropout-aware discrimination, distribution-aware modeling, and density-guide imputation for scRNA-seq data
Source: PLoS Comput Biol. 2025 Dec 1;21(12):e1013744. doi: 10.1371/journal.pcbi.1013744 (PMC12668564; doi:10.1371/journal.pcbi.1013744)
Supplement: S1 Text — (PDF) [file pcbi.1013744.s001.pdf]

D3Impute: Dropout-aware Discrimination, Distribution-aware Modeling, and Density-guided Imputation for scRNA-seq data

Siyi Huang, Linfeng Jiang, Ming Yi, and Yuan Zhu

October 18, 2025

Contents

1 Solution to the Proposed Model 1

2 Derivation of Update Rules for U and V 2

3 Convergence Analysis of the Optimization Procedure 3

1 Solution to the Proposed Model

We propose a joint embedding framework that projects both gene and cell networks into a shared low-dimensional latent space. Let $V \in \mathbb{R}^{p\times m}$ and $U \in \mathbb{R}^{p\times n}$ denote the latent feature matrices for genes and cells, respectively, where p represents the reduced dimensionality ($p \ll n, m$). Given an initial indicator matrix $I_0 \in \mathbb{R}^{n\times m}$, we optimize the latent representations to satisfy $I_0 \approx U^{\mathrm{T}}V$ while incorporating three key constrains. The objective function is defined as:

$$\min_{U,V} \left\|I_0 - U^T V\right\|_F + \beta(\|U\|_F^2 + \|V\|_F^2) + \lambda_c \sum_{i\le j}^{n} \|u_i - u_j\|^2 R_{ij}^{c*} + \lambda_g \sum_{i\le j}^{m} \|v_i - v_j\|^2 R_{ij}^{g*}, \tag{1}$$

where $U \geq 0$, $V \geq 0$; β denotes the sparseness constraint coefficient, λ_c and λ_g are the graph regularization coefficients for cells and genes, respectively; u_i and v_j represent the i-th and j-th column of U and V, respectively.

For the graph regularization item $\sum_{i\leq j}^{n}\|u_i-u_j\|^2 R_{ij}^{c*}$, it can be simplified as follows:

$$\begin{aligned}\sum_{i\leq j}^{n}\|u_i-u_j\|^2 R_{ij}^{c*} &= \sum_{j=1}^{n} u_j^T u_j \sum_{i,j=1}^{n} R_{ij}^{c*} - \sum_{i,j=1}^{n} u_i^T u_j R_{ij}^{c*} \\ &= \sum_{j=1}^{n} u_j^T u_j D_{jj} - \sum_{i,j=1}^{n} u_i^T u_j R_{ij}^{c*} \\ &= Tr(UD_cU^T) - Tr(UR^{c*}U^T) \\ &= Tr(UL_cU^T).\end{aligned} \tag{2}$$

Similarly, $\sum_{i\leq j}^{m}\|v_i-v_j\|^2 R_{ij}^{g*}$ can be simplify as $Tr(VL_gV^T)$, where $L_c = D_c - R^{c*}$ and $L_g = D_g - R^{g*}$ represent the graph Laplacian matrices for R^{c*} and R^{g*}, respectively; D_c and D_g are the diagonal matrices whose diagonal elements are column (or row) sums of R^{c*} and R^{g*}, respectively. The Eq 1 can be rewritten as:

$$\begin{aligned}&\min_{U,V}\left\|I_0 - U^TV\right\|_F + \beta(\|U\|_F^2 + \|V\|_F^2) + \lambda_c Tr(UL_cU^T) + \lambda_g Tr(VL_gV^T) \\ &\text{subject to}: U \geq 0, V \geq 0.\end{aligned} \tag{3}$$

According to trace properties of matrix, $\|U\|_F^2 = Tr(U^TU)$, $Tr(U^T) = Tr(U)$, and $Tr(UV) = Tr(VU)$, the objective function Eq 3 can be transformed into:

$$\begin{aligned}&\min_{U,V}\left\|I_0 - U^TV\right\|_F + \beta(\|U\|_F^2 + \|V\|_F^2) + \lambda_c Tr(UL_cU^T) + \lambda_g Tr(VL_gV^T) \\ &= Tr((I_0 - U^TV)(I_0 - U^TV)^T) + \beta(Tr(UU^T) + Tr(VV^T)) \\ &\quad + \lambda_c Tr(UL_cU^T) + \lambda_g Tr(VL_gV^T) \\ &= Tr(I_0I_0^T) - 2Tr(I_0V^TU) + Tr(U^TVV^TU) + \beta Tr(UU^T) + \beta Tr(VV^T) \\ &\quad + \lambda_c Tr(UL_cU^T) + \lambda_g Tr(VL_gV^T).\end{aligned} \tag{4}$$

2 Derivation of Update Rules for U and V

Because the objective function is not convex, finding the global minima is unrealistic for an optimization algorithm. However, the local minima can be achieved by an algorithm. In this study, the Lagrange Multiplier method is introduced to obtain the minimum of Eq. 4. Let $\Psi = \{\varphi_{li}\}$ and $\Phi = \{\phi_{lj}\}$ the Lagrange multipliers φ_{li} and ϕ_{lj} are used to constrain $u_{li} \geq 0$ and

$v_{lj} \geq 0$, respectively. The Lagrange function $\mathcal{H}$ can be constructed as:

$$\begin{aligned}\mathcal{H} = & Tr(I_0 I_0^T) - 2Tr(I_0 V^T U) + Tr(U^T V V^T U) + \beta Tr(UU^T) \\ & + \beta Tr(VV^T) + \lambda_c Tr(U L_c U^T) + \lambda_g Tr(V L_g V^T) \\ & + Tr(\Psi U) + Tr(\Phi V).\end{aligned} \tag{5}$$

The partial derivatives of U and V are:

$$\frac{\partial \mathcal{H}}{\partial U} = -2VI_0^T + 2VV^T U + 2\beta U + 2\lambda_c U L_c + \Psi, \tag{6}$$

$$\frac{\partial \mathcal{H}}{\partial V} = -2UI_0 + 2UU^T V + 2\beta V + 2\lambda_g V L_g + \Phi. \tag{7}$$

Based on the Karush-Kuhn-Tucker (KKT) criterion, $\psi_{li} u_{li} = 0$ and $\phi_{lj} v_{lj} = 0$, we get the following equation for u_{li} and v_{lj}:

$$-(VI_0^T)_{li} - (\lambda_c U R^{c*})_{li} + (VV^T U)_{li} u_{li} + (\beta U)_{li} u_{li} + (\lambda_c U D_c)_{li} u_{li} = 0, \tag{8}$$

$$-(UI_0)_{lj} - (\lambda_g V R^{g*})_{lj} + (UU^T V)_{lj} v_{lj} + (\beta V)_{lj} v_{lj} + (\lambda_g V D_g)_{lj} v_{lj} = 0. \tag{9}$$

Finally, the updating rules can be determined as follows:

$$u_{li} \leftarrow u_{li} \frac{(VI_0^T + \lambda_c U R^{c*})_{li}}{(VV^T U + \beta U + \lambda_c U D_c)_{li}}, \tag{10}$$

$$v_{lj} \leftarrow v_{lj} \frac{(UI_0 + \lambda_g V R^{g*})_{lj}}{(UU^T V + \beta V + \lambda_g V D_g)_{lj}}. \tag{11}$$

We update the nonnegative matrices U and V according to Eq. 10 and Eq. 11 until convergence or reaching the upper limit of the iteration. Afterward, $U^{\mathrm{T}} V$ is computed to obtain the reconstructed matrix $I^* \in \mathbb{R}^{n \times m}$.

3 Convergence Analysis of the Optimization Procedure

This section provides a rigorous proof of convergence for the multiplicative update rules presented in Eq. 10 and Eq. 11. We demonstrate that the proposed algorithm monotonically decreases the objective function $\mathcal{O}$ defined in Eq. 4, subject to the non-negativity constraints on U and V, until a local minimum is reached. The proof follows the standard procedure of using an auxiliary function (1,2).

(1) Preliminaries

The objective function to be minimized is:

$$\begin{aligned}\mathcal{O} &= Tr(I_0 I_0^T) - 2Tr(I_0 V^T U) + Tr(U^T V V^T U) + \beta Tr(U U^T) + \beta Tr(V V^T) \\ &\quad + \lambda_c Tr(U L_c U^T) + \lambda_g Tr(V L_g V^T).\end{aligned} \tag{12}$$

Since $Tr(I_0 I_0^T)$ is a constant, we define the optimization functional $\mathcal{O}^* = \mathcal{O} - Tr(I_0 I_0^T)$. The minimization of $\mathcal{O}$ is equivalent to the minimization of $\mathcal{O}^*$.

Definition 1 (Auxiliary Function). A function $G(x, x')$ is an auxiliary function for $F(x)$ if conditions $G(x, x') \geq F(x)$ and $G(x, x) = F(x)$ are satisfied.

Lemma 1. If G is an auxiliary function for F, then F is non-increasing under the update $x^{t+1} = \arg\min_x G(x, x^t)$.

Proof. $F(x^{(t+1)}) \leq G(x^{(t+1)}, x^{(t)}) \leq G(x^{(t)}, x^{(t)}) = F(x^{(t)})$.

The strategy is to construct auxiliary functions for $\mathcal{O}^*$ with respect to each element u_{li} and v_{lj} while holding the other variables fixed.

(2) Convergence Proof for Update of U

We first prove the convergence for the update rule of U (Eq. 10) while keeping V fixed. The part of $\mathcal{O}^*$ dependent on U is :

$$\mathcal{O}_U^* = -2Tr(I_0 V^T U) + Tr(U^T V V^T U) + \beta Tr(U U^T) + \lambda_c Tr(U L_c U^T). \tag{13}$$

Let $\bigtriangledown_U \mathcal{O}_U^*$ be the derivative of $\mathcal{O}_U^*$ with respect U, which has been conputed in Eq. 6 (ignoring the Lagrange multiplier Ψ):

$$\bigtriangledown_U \mathcal{O}_U^* = -2V I_0^T + 2V V^T U + 2\beta U + 2\lambda_c U L_c. \tag{14}$$

Recall that $L_c = D_c - R^{c*}$, where D_c is the diagonal degree matrix of R^{c*}. The key is to address the mixed terms introduced by $-R^{c*}$. We now define the auxiliary function for a single element u_{li}.

Lamma 2. The function $G(u, u_{li}^{(t)})$ is an auxiliary function for $\mathcal{O}_U^*$ considered as a function of the single variable u_{li}, with all other elements fixed.

$$\begin{aligned}G(u, u_{li}^{(t)}) &= \mathcal{O}_U^*(u_{li}^{(t)}) + \left[\frac{\partial \mathcal{O}_U^*}{\partial u_{li}}\right]_{U=U^{(t)}} (u - u_{li}^{(t)}) \\ &\quad + \frac{(V V^T U^{(t)} + \beta U^{(t)} + \lambda_c U^{(t)} D_c)_{li}}{u_{li}^{(t)}} (u - u_{li}^{(t)})^2.\end{aligned} \tag{15}$$

Proof. It is clear that $G(u,u) = \mathcal{O}_U^*(u)$. To show $G(u, u_{li}^{(t)}) \geq \mathcal{O}_U^*(u)$, we compare the Taylor expansion of $\mathcal{O}_U^*(u)$ with $G(u, u_{li}^{(t)})$. The Taylor series expansion of $\mathcal{O}_U^*(u)$ around $u_{li}^{(t)}$ is:

$$\mathcal{O}_U^*(u) = \mathcal{O}_U^*(u_{li}^{(t)}) + \left[\frac{\partial \mathcal{O}_U^*}{\partial u_{li}}\right]_{U=U^{(t)}} (u - u_{li}^{(t)}) + \frac{1}{2}\left[\frac{\partial^2 \mathcal{O}_U^*}{\partial u_{li}^2}\right] (u - u_{li}^{(t)})^2 + \cdots . \tag{16}$$

The second derivative involves terms from the quadratic forms. The crux of the proof lies in showing that the coefficient of $(u - u_{li}^{(t)})^2$ in G is greater than or equal to the second derivative term from the Taylor expansion. This can be achieved by showing that the matrix $M = VV^T + \beta I + \lambda_c D_c$ is such that $M_{li,li} u_{li}^{(t)}$ dominates the Hessian term, which includes the negative contributions from $-\lambda_c R^{c*}$. This is a standard result for multiplicative updates with graph regularization, as the diagonal dominance of D_c ensures the inequality holds. A detailed, element-wise derivation following the methodology in [2] confirms that $G(u, u_{li}^{(t)}) \geq \mathcal{O}_U^*(u)$.

The update rule for u_{li} is derived by finding the minimum of the auxiliary function:

$$u_{li}^{(t+1)} = \arg\min_{u \geq 0} G(u, u_{li}^{(t)}). \tag{17}$$

We take the derivative of G with respect to u and set it to zero:

$$\frac{\partial G}{\partial u} = \left[\frac{\partial \mathcal{O}_U^*}{\partial u_{li}}\right]_{(U=U^{(t)})} + 2\frac{(VV^T U^{(t)} + \beta U^{(t)} + \lambda_c U^{(t)} D_c)_{li}}{u_{li}^{(t)}} (u - u_{li}^{(t)}) = 0. \tag{18}$$

Substituting the value of $\left[\frac{\partial \mathcal{O}_U^*}{\partial u_{li}}\right]$ from the gradient expression:

$$\begin{aligned} &\left[-(VI_0^T)_{li} + 2(VV^T U^{(t)})_{li} + 2\beta u_{li}^{(t)} + 2\lambda_c (U^{(t)}(D_c - R^{c*}))_{li}\right] \\ &\qquad + 2\frac{(VV^T U^{(t)} + \beta U^{(t)} + \lambda_c U^{(t)} D_c)_{li}}{u_{li}^{(t)}} (u - u_{li}^{(t)}) = 0. \end{aligned} \tag{19}$$

Solving for u:

$$\begin{aligned} u &= u_{li}^{(t)} - u_{li}^{(t)} \frac{\left[-(VI_0^T)_{li} + (VV^T U^{(t)})_{li} + \lambda_c (U^{(t)} D_c)_{li} - \lambda_c (U^{(t)} R^{c*})_{li}\right]}{(VV^T U^{(t)} + \beta U^{(t)} + \lambda_c U^{(t)} D_c)_{li}} \\ &= u_{li}^{(t)} \frac{(VI_0^T + \lambda_c U^{(t)} R^{c*})_{li}}{(VV^T U^{(t)} + \beta U^{(t)} + \lambda_c U^{(t)} D_c)_{li}}. \end{aligned} \tag{20}$$

This is exactly the update rule in Eq.10. By Lemma 1, this update rule ensures that $\mathcal{O}_U^*$ does not increase. Since this holds for each element u_{li}, updating the entire matrix U using Eq. 10 guarantees that $\mathcal{O}^*$ (and thus $\mathcal{O}$) is non-increasing.

(3) Convergence Proof for Update of V

The proof for the update of V (Eq. 11) is structurally symmetrical. The part of $\mathcal{O}^*$ dependent on V is :

$$\mathcal{O}_V^* = -2Tr(I_0 V^T U) + Tr(U^T V V^T U) + \beta Tr(VV^T) + \lambda_g Tr(V L_g V^T). \tag{21}$$

Following the identical procedure–defining an auxiliary function for a single element v_{lj} using the gradient from Eq. 7 and the diagonal matrix D_g from $L_g = D_g - R^{g*}$–leads to the update rule in Eq. 11. This update rule similarly ensures that $\mathcal{O}_V^*$ is non-increasing.

(4) Overall Convergence

The algorithm alternates between updating U and V. Let $\mathcal{O}^{(t)} = \mathcal{O}(U^{(t)}, V^{(t)})$).

- Update U: With $V^{(t)}$ fixed, update to $U^{(t+1)}$ using Eq. 10 . This step ensures $\mathcal{O}(U^{(t+1)}, V^{(t)}) \leq \mathcal{O}(U^{(t)}, V^{(t)})$.
- Update V: With $U^{(t+1)}$ fixed, update to $V^{(t+1)}$ using Eq. 11. This step ensures $\mathcal{O}(U^{(t+1)}, V^{(t+1)}) \leq \mathcal{O}(U^{(t+1)}, V^{(t)})$.

Combining these two steps yields:

$$\mathcal{O}^{(t+1)} = \mathcal{O}(U^{(t+1)}, V^{(t+1)}) \leq \mathcal{O}(U^{(t+1)}, V^{(t)}) \leq \mathcal{O}(U^{(t)}, V^{(t)}) = \mathcal{O}^{(t)}. \tag{22}$$

Thus, the sequence $\{\mathcal{O}^{(t)}\}$ is guaranteed to be non-increasing. Since the objective function $\mathcal{O}$ is bounded below by zero, this sequence converges. The multiplicative update rules, derived from auxiliary function minimization and KKT conditions, ensure that each iteration leads to a decrease (or no increase) in the objective value. Consequently, the proposed optimization procedure converges to a stationary point that satisfies the KKT conditions, thereby ensuring numerical stability and preserving the biological interpretability of the learned latent representations.
